# Exploiting the *ZIP4* homologue within the wheat *Ph1* locus has identified two lines exhibiting homoeologous crossover in wheat-wild relative hybrids

**DOI:** 10.1007/s11032-017-0700-2

**Published:** 2017-07-18

**Authors:** María-Dolores Rey, Azahara C. Martín, Janet Higgins, David Swarbreck, Cristobal Uauy, Peter Shaw, Graham Moore

**Affiliations:** 1 John Innes Centre, Norwich Research Park, Norwich, NR4 7UH UK; 2 Earlham Institute, Norwich Research Park, Norwich, NR4 7UZ UK

**Keywords:** Wheat, *Ph1*, *ZIP4*, Homoeologues, Synapsis, Crossover

## Abstract

Despite possessing related ancestral genomes, hexaploid wheat behaves as a diploid during meiosis. The wheat *Ph1* locus promotes accurate synapsis and crossover of homologous chromosomes. Interspecific hybrids between wheat and wild relatives are exploited by breeders to introgress important traits from wild relatives into wheat, although in hybrids between hexaploid wheat and wild relatives, which possess only homoeologues, crossovers do not take place during meiosis at metaphase I. However, in hybrids between *Ph1* deletion mutants and wild relatives, crossovers do take place. A single *Ph1* deletion (*ph1b*) mutant has been exploited for the last 40 years for this activity. We show here that chemically induced mutant lines, selected for a mutation in *TaZIP4-B2* within the *Ph1* locus, exhibit high levels of homoeologous crossovers when crossed with wild relatives. *Tazip4-B2* mutant lines may be more stable over multiple generations, as multivalents causing accumulation of chromosome translocations are less frequent. Exploitation of such *Tazip4-B2* mutants, rather than mutants with whole *Ph1* locus deletions, may therefore improve introgression of wild relative chromosome segments into wheat.

**Electronic supplementary material:**

The online version of this article (doi:10.1007/s11032-017-0700-2) contains supplementary material, which is available to authorized users.

## Introduction

Hexaploid bread wheat (*Triticum aestivum*) is composed of three related ancestral genomes (A, B and D), each containing seven identical (homologous) chromosome pairs (homologue pairs 1–7). Each homologue pair has a corresponding related homoeologous chromosome pair, possessing similar gene order and content, within each of the other two genomes. Despite the similarity between homoeologues, wheat behaves as a diploid at meiosis, with synapsis and crossovers (COs) only occurring between homologous chromosomes, rather than between homoeologous chromosomes (for example, 1A only pairs with 1A but not with either 1B or 1D, holding true for all seven chromosome groups). This diploid-like behaviour is predominantly controlled by *Ph1*, a dominant locus on chromosome 5B (Riley and Chapman 1958; Sears and Okamoto 1958).

The *Ph1* locus was defined as a deletion phenotype, first described by scoring meiosis in wheat hybrids lacking the whole 5B chromosome (Riley and Chapman 1958; Sears and Okamoto 1958). The effect of deleting the *Ph1* locus in wheat itself is to reduce the level of ring bivalents (and increase rod bivalent levels) at metaphase I in all meiocytes, while inducing a low level of multivalents and univalents in some meiocytes (Roberts et al. 1999). Sexual hybridisation between wheat and wild relatives (for example, rye or *Aegilops variabilis*) produces interspecific hybrids, containing haploid sets of wheat and wild relative homoeologous chromosomes, but exhibiting virtually no COs during meiosis. However, in *Ph1* deleted wheat-rye hybrids, an average of 7 COs per cell is observed (Sears 1977; Dhaliwal et al. 1977). These *Ph1* deleted hybrids have been used to introgress important traits from wild relatives into wheat. Thus, the absence of the *Ph1* locus is scored as a reduction in homologous crossovers at metaphase I in wheat meiocytes and an increase in homoeologous crossovers at metaphase I in wheat-wild relative meiocytes.

Ethylmethane sulphonate (EMS) treatment failed to produce mutants exhibiting the full *Ph1* deletion phenotype because of the difficulty with the phenotype screen. This suggests that the *Ph1* phenotype most likely results from activity of more than a single gene (Griffiths et al. 2006). However, exploitation of smaller chromosome 5B deletions later characterised and localised the *Ph1* locus to a 2.5 MB region on chromosome 5B (Griffiths et al. 2006). More recently, a metaphase I expressing gene within the 2.5 MB region (corresponding to the wheat gene EST BE498862 and the rice gene Os9g30320), and termed *C-Ph1* by the authors, has been proposed as the putative *Ph1* gene (Bhullar et al. 2014). The assertion was based on the observation of clumping or multiple chromosome associations in wheat meiocytes at metaphase I, with increased plant sterility, using a VIG-based approach rather than deletion analysis. However, previous studies have already shown that the rice homologue Os9g30320 is actually a tapetal cell gene (Jeon et al. 1999). In addition, a wheat paralogue of the *C-Ph1* gene (termed Raftin1) has also previously been characterised as a tapetal cell gene (Wang et al. 2003). The expression peak of tapetal cell genes occurs when the tapetum fully forms around the metaphase I meiocytes. Disruption of these genes results in stressed meiocytes, chromosome clumping and male sterility, hence the previous filing of patents for the exploitation of these genes to generate male sterile lines (Patents WO2000026389 A3 and US20040060084). Moreover, two previously described deletions within the 2.5 MB region, also encompassing *C-Ph1*, did not exhibit a *Ph1* mutant phenotype (Al-Kaff et al. 2008). Therefore, either the phenotype of these previously described deletions has been incorrectly scored, or the phenotype of the VIG approach was due to off-target effects (such as disruption of homoeologous and paralogous tapetal cell gene function). The observed VIG phenotype was also noted to be more extreme than when the whole 5B chromosome is deleted (Bhullar et al. 2014).

Deletion analysis defines the *Ph1* locus to the part of the 2.5 MB region containing a duplicated chromosome 3B segment carrying heterochromatin and *TaZIP4-B2* (originally termed *Hyp3*, UniProtKB-Q2L3T5), inserted into a cluster of *CDK2-like* genes interspersed with methyl transferase genes (originally termed *SpG*, UniProtKB-Q2L3W3) (Griffiths et al. 2006; Al-Kaff et al. 2008; Martín et al. 2017). The contribution of these genes to the *Ph1* phenotype is not known; therefore, the effect of these genes on synapsis and crossover required further analysis. Synapsis is a process early in meiosis by which homologues intimately align with each other, forming bivalents held together by a proteinaceous structure named synaptonemal complex (SC). Ultimately, the SC is degraded, so that the bivalents are only held together by chiasmata or COs at metaphase I, allowing their correct segregation. Recently, *Ph1* has been shown to have a dual effect on synapsis and CO formation in wheat (Martín et al. 2014; Martín et al. 2017). The effect on synapsis occurs during the telomere bouquet stage, when *Ph1* promotes more efficient homologous synapsis, thereby reducing the chance of homoeologous synapsis (Martín et al. 2017). The effect on CO formation happens later in meiosis, when *Ph1* prevents MLH1 sites (Double Holliday Junctions marked to become COs) on synapsed homoeologues from becoming COs. However, the molecular mechanism of how the *Ph1* locus affects both synapsis and CO is not currently known.

A single *Ph1* deletion mutant, developed in hexaploid wheat cv. Chinese Spring (CS) (CS *ph1b)*, has been used by breeding programmes worldwide to introgress wild relative chromosome segments into wheat. A recent programme has successfully introgressed hundreds of wild relative chromosome segments into wheat, exploiting CS *ph1b* (King et al. 2016). The segments were identified through a combination of both DNA array and cytological-based approaches. Although CS *ph1b* has been successfully used in breeding programmes, the CS *ph1b* mutant is reported to accumulate extensive rearrangements reducing fertility (Sánchez-Morán et al. 2001), due to homoeologous synapsis and COs, as visualised by the occurrence of multivalents at metaphase I during meiosis. It would therefore be useful to identify novel wheat *Ph1* mutant lines, with reduced homoeologous synapsis and CO at meiosis, but which do exhibit homoeologous COs in hybrids with wild relatives. As described previously, the *Ph1* locus, which affects both synapsis and CO, is a complex cluster of *CDK2*-like and methyl transferase genes containing a *ZIP4* paralogue. It has been proposed that *Ph1*’s effect on synapsis is connected to altered Histone H1 CDK2-dependent phosphorylation in the presence and absence of *Ph1*. Altered phosphorylation affects chromatin structure and delays premeiotic replication, subsequently affecting homologue synapsis, thus allowing homoeologous synapsis to take place (Greer et al. 2012; Martín et al. 2017). Lines carrying mutations in the *Ph1CDK2*-like homologue in Arabidopsis also exhibit reduced synapsis under specific conditions, suggesting a role for these genes in efficient synapsis (Zheng et al. 2014). We have also previously proposed that the effect of *CDK2*-like genes on chromatin structure not only affects synapsis, but might also affect the resolution of Double Holliday Junctions (marked by MLH1) as COs (Greer et al. 2012). Okadaic acid treatment affects chromatin structure and can induce homoeologous CO in wheat-wild relative hybrids (Knight et al. 2010). However, given that the locus contains multiple copies of the *CDK2*-like and methyltransferase genes, it would be a complex and laborious study to identify EMS mutants within these genes and combine possible mutations. The transfer of multiple mutated genes into different elite genetic backgrounds by breeders, for subsequent crossing with wild relatives, would also be laborious. Moreover, as previously indicated, we want to identify wheat mutant lines which exhibit reduced homoeologous synapsis or multivalents at metaphase I; so, the *CDK2*-like genes would not be the initial candidates for such an approach.

Although there are *ZIP4* homologues on group 3 chromosomes, the *ZIP4* paralogue (*TaZIP4-B2*) within the *Ph1* locus on chromosome 5B is single copy, compared to the *CDK2*-like and methyl transferase gene cluster. Moreover, *ZIP4* has been shown to have a major effect on homologous COs, but not on synapsis, in both Arabidopsis and rice (Chelysheva et al. 2007; Shen et al. 2012). Knockouts of this gene in diploids usually result in sterility, as elimination of homologous COs leads to metaphase I pairing failure and incorrect segregation. We therefore assessed whether the selection and scoring of *Tazip4-B2* EMS mutants would identify wheat lines with reduced homologous CO and minimal homoeologous synapsis and CO (as observed by the occurrence of multivalents at metaphase I), but which exhibit homoeologous COs in hybrids with wild-relatives. We describe the identification of two such lines through this approach.

## Materials and methods

### Plant material

Plant material used in this study included the following: wild-type hexaploid wheat (*Triticum aestivum* cv. Chinese Spring and cv. Cadenza); a Chinese Spring mutant lacking the *Ph1* locus (*ph1b*); two *Tazip4-B2* mutant Cadenza lines (Cadenza1691 and Cadenza0348; M_4_ generation (Supplementary Table 2)); hexaploid wheat—*Aegilops variabilis* hybrids—crosses between hexaploid wheat (*T. aestivum* cv. Cadenza) and *Ae. variabilis* (2n = 4× = 28)—using either wild-type Cadenza or *Tazip4*-B2 mutant lines.

Both for the RNA-seq analysis and for meiotic studies, the seedlings were vernalised for 3 weeks at 8 °C and then transferred to a controlled environmental room until meiosis under the following growth conditions: 16 h/8 h, light/dark photoperiod at 20 °C day and 15 °C night, with 70% humidity. Tillers were harvested after 6 to 7 weeks, when the flag leaf was starting to emerge, and anthers collected. For each dissected floret, one of the three synchronised anthers was squashed in 45% acetic acid in water and assigned to each meiotic stage by observation under a LEICA DM2000 microscope (LeicaMicrosystems, http://www.leica-microsystems.com/). The two remaining anthers were either frozen in liquid nitrogen and stored at −80 °C for RNA-seq analysis or fixed in 100% ethanol/acetic acid 3:1 (*v*/*v*) for cytological analysis of meiocytes.

### RNA-seq experiments

#### Sample preparation

Anthers from wild-type wheat (WT) and wheat lacking the *Ph1* locus (*ph1b* deletion) were harvested as described in the “Plant material” section. Anthers at late leptotene-early zygotene stage were later harvested into RNA later (Ambion, Austin, TX). The anthers from three plants of each genotype were pooled in a 1.5-ml Eppendorf until reaching 200 to 400 anthers. Once sufficient anthers had been collected, the material was disrupted using a pestle, centrifuged to eliminate the RNA and then homogenised using QIAshredder spin columns (Qiagen, Hilden, Germany). RNA extraction was performed using a miRNeasy Micro Kit (Qiagen, Hilden, Germany) according to the manufacturer’s instructions. This protocol allows purification of a separate miRNA-enriched fraction (used for further analysis) and the total RNA fraction (˃200 nt) used in this study. This process was repeated to obtain three biological samples of each genotype.

#### RNA-seq library preparation and sequencing

One microgram of RNA was purified to extract mRNA with a poly-A pull down using biotin beads. A total of six libraries were constructed using the NEXTflex™ Rapid Directional RNA-Seq Kit (Bioo Scientific Corporation, Austin, Texas, USA) with the NEXTflex™ DNA Barcodes–48 (Bioo Scientific Corporation, Austin, Texas, USA) diluted to 6 μM. The library preparation involved an initial QC of the RNA using Qubit DNA (Life technologies, CA, Carlsbad) and RNA (Life technologies, CA, Carlsbad) assays as well as a quality check using the PerkinElmer GX with the RNA assay (PerkinElmer Life and Analytical Sciences, Inc., Waltham, MA, USA). The constructed stranded RNA libraries were normalised and equimolar pooled into one final pool of 5.5 nM using elution buffer (Qiagen, Hilden, Germany, Hilden, Germany). The library pool was diluted to 2 nM with NaOH, and 5 μl was transferred into 995 μl HT1 (Illumina) to give a final concentration of 10 pM. Diluted library pool of 120 μl was then transferred into a 200-μl strip tube, spiked with 1% PhiX Control v3 and placed on ice before loading onto the Illumina cBot. The flow cell was clustered using HiSeq PE Cluster Kit v4, utilising the Illumina PE_HiSeq_Cluster_Kit_V4_cBot_recipe_V9.0 method on the Illumina cBot. Following the clustering procedure, the flow cell was loaded onto the Illumina HiSeq2500 instrument following the manufacturer’s instructions. The sequencing chemistry used was HiSeq SBS Kit v4 with HiSeq Control Software 2.2.58 and RTA 1.18.64. The library pool was run in a single lane for 125 cycles of each paired end read. Reads in bcl format were demultiplexed based on the 6 bp Illumina index by CASAVA 1.8, allowing for a one base-pair mismatch per library and converted to FASTQ format by bcl2fastq.

#### RNA-seq data processing

The raw reads were processed using SortMeRNA v2.0 (Kopylova et al. 2012) to remove rRNA reads. The non-rRNA reads were then trimmed using Trim Galore v0.4.1 (http://www.bioinformatics.babraham.ac.uk/projects/trim_galore/) to remove adaptor sequences and low-quality reads (-q 20—length 80—stringency 3). A total of 273,739 transcripts (Triticum_aestivum_CS42_TGACv1_scaffold.annotation) were quantified using kallisto v0.43.0 (Bray et al. 2016). The index was built using a k-mer length of 31; then, Kallisto quant was run using the following options -b 100—rf-stranded. Transcript abundance was obtained as Transcripts Per Million (TPM) for each gene.

### Cytological analysis and image processing

A total of five plants per line were examined in a randomised block design. Generally, 2–3 tillers in each plant were analysed to identify meiocytes at meiotic metaphase I. Anthers from *Tazip4-B2* Cadenza mutant lines, wild-type Cadenza, *Tazip4-B2* mutant line-*Ae variabilis* hybrids, and Cadenza-*Ae. variabilis* hybrids were harvested as described in the “Plant material” section. Cytological analysis of meiocytes was performed using Feulgen reagent as previously described (Sharma and Sharma 2014). Images were collected using a LEICA DM2000 microscope (LeicaMicrosystems, http://www.leica-microsystems.com/), equipped with a Leica DFC450 camera and controlled by LAS v4.4 system software (Leica Biosystems, Wetzlar, Germany). Images were processed using Adobe Photoshop CS5 (Adobe Systems Incorporated, US) extended version 12.0 × 64.

### Nucleotide analysis

The regions of each mutation in both mutant Cadenza lines were sequenced to confirm the existence of either missense or nonsense mutations in Cadenza1961 and Cadeza0348, respectively. Wheat leaf tissues from wild type Cadenza and mutant Cadenza lines were harvested at growth stages 3–4 (Feekes scale). DNA was extracted using the CTAB method (Murray and Thompson 1980). Two pairs of primers were designed using the Primer3plus software (Untergasser et al. 2007) based on the *ZIP4* sequence called TRIAE_CS42_5BL_TGACv1_404600_AA1305800 (http://plants.ensembl.org/Triticum_aestivum/Info/Index). The primers used were as follows: forward primer: 5′GCCGCCATGACGATCTCCGAG3′ and reverse primer: 5′GGACGCGAGGGACGCGAG3′ for Cadenza1691 and forward primer: 5′GTGTTCCTAATGCTCACAACTC3′ and reserve primer: 5′ACCAGACATACTTGTGCTTGGT3′ for Cadenza0348. PCR amplification was performed using MyFi Polymerase (Bioline Tauton, MA, USA), according to the manufacturer’s instructions. The primers were amplified as follows: 3 min 95 °C, 35 cycles of 15 s at 95 °C, 15 s at 58 °C and 30 s at 72 °C. PCR products were resolved on 2% agarose gels in 1× TBE and stained with ethidium bromide and visualised under UV light. PCR products were purified using Qiaquick PCR Purification kits (Qiagen, Hilden, Germany) and cloned using a p-GEM T easy vector kit (Promega, Madison, Wisconsin, USA). The ligation mixture was used to transform *Escherichia coli* DH5a, and transformants were selected on LB agar containing ampicillin (100 mg/ml) (Sigma, St. Louis, MO, USA), isopropyl β-D-1-thiogalactopyranoside (IPTG, 100 mM) (Sigma, St. Louis, MO, USA) and 5-bromo-4-chloro-3-indolyl β-D-galactopyranoside (X-Gal, 20 mg/ml) (Sigma, St. Louis, MO, USA). The PCR fragments were isolated using QIAprep Spin Miniprep kit (Qiagen, Hilden, Germany). All kits were used as described in the manufacturer’s instructions. Sequencing was carried out by the Eurofins Company. Alignment of sequences was carried out using Clustal Omega software (http://www.ebi.ac.uk/Tools/msa/clustalo/).

### Statistical analyses

Statistical analyses were performed using STATISTIX 10.0 software (Analytical Software, Tallahassee, FL, USA). Both *Tazip4-B2* mutant lines-*Ae. varibialis* hybrids and *Tazip4*-B2 mutant lines were analysed by the Kruskal–Wallis test (nonparametric one-way analysis of variance). Means were separated using the Dunn’s test with a probability level of 0.05.

## Results and discussion

### *TaZIP4-B2* expression

**Fig. 1:**
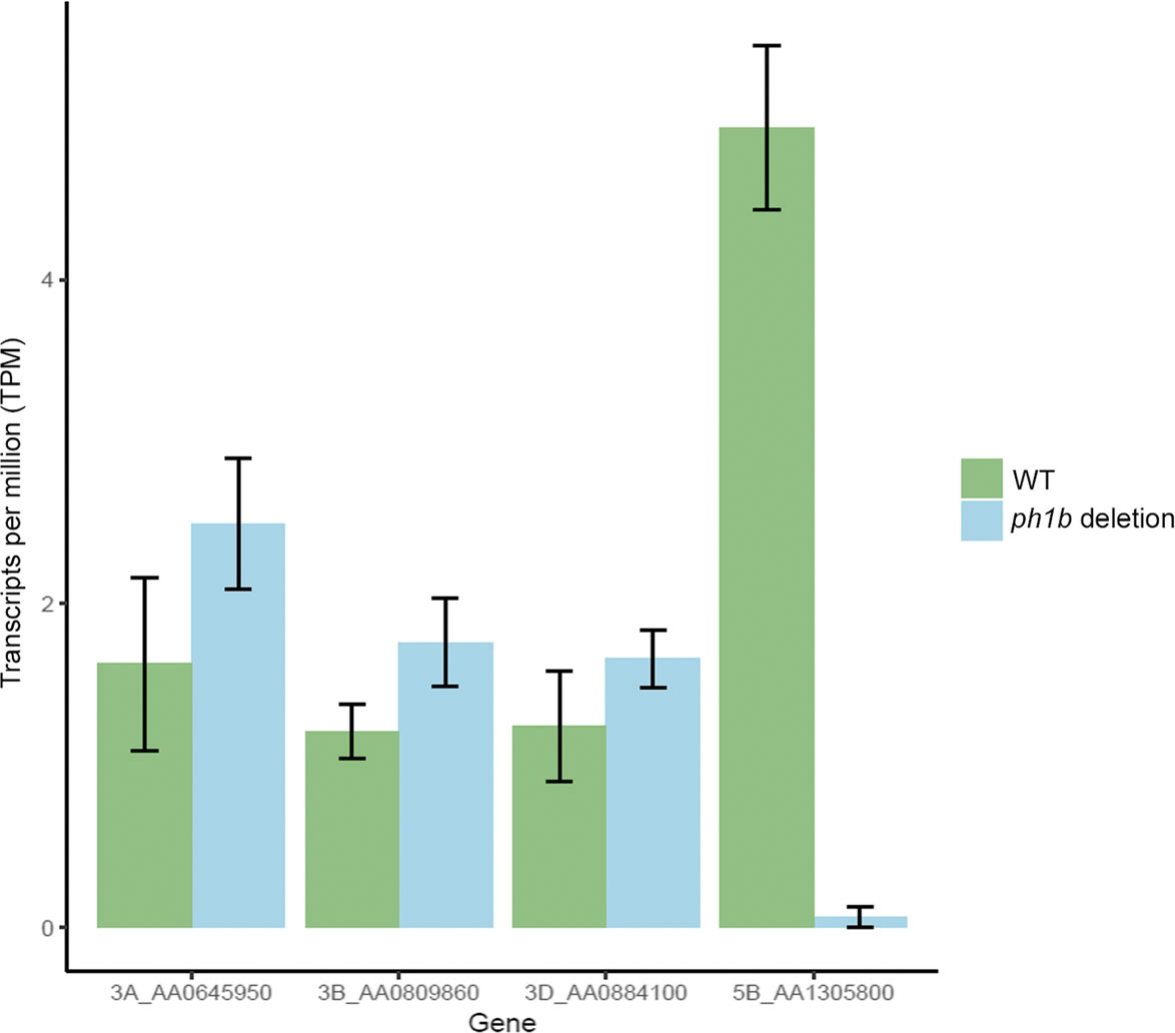
Relative expression of *ZIP4* homologues in *Triticum aestivum* cv. Chinese Spring in presence (WT) and in absence (*ph1b* deletion) of the *Ph1* locus obtained by RNA-seq analysis

In hexaploid wheat, *ZIP4* homologues are located within the *Ph1* locus on 5B, and also on chromosomes 3A, 3B and 3D. Before undertaking the targeted induced lesion in genomes (TILLING) mutant analysis, we assessed the expression of *TaZIP4-B2* to confirm that the *TaZIP4-B2* gene within the *Ph1* locus is expressed during meiosis; that it has a higher level of expression than the *ZIP4* homologues present on chromosome group 3; and finally, that *Ph1* deletion significantly reduces overall *ZIP4* expression. At the coding DNA sequence and amino acid levels, *TaZIP4-B2* (AA1305800.1) showed 95.3 and 89.2% similarity to *TaZIP4-B1* (AA0809860.1), 94.1 and 87.5% similarity to *TaZIP4-A1* (AA0645950.1) and 94.4 and 87.2% similarity to *TaZIP4-D1* (AA0884100.1), respectively (Supplementary Fig. 1). To compare the relative expression of these *ZIP4* homologues on chromosomes 3A, 3B, 3D and 5B, RNA samples were collected from anthers of hexaploid wheat (*Triticum aestivum* cv. Chinese Spring) (WT) and the *Ph1* deletion mutant (*ph1b*) at the late leptotene-early zygotene stage, and six libraries prepared for the RNA-seq study. RNA-seq analysis showed that *TaZIP4-B2* exhibited a higher level of expression than the *ZIP4* homologues on homoeologous group 3 chromosomes (Fig. 1; Supplementary Fig. 1). Moreover, *TaZIP4-B2* also showed three splice variants (Supplementary Fig. 2) in contrast to homoeologous group 3 chromosome *ZIP4* homologues. One of these splice variants (splice variant 1) accounted for 97% of the *TaZIP4-B2* transcripts. As expected, when the *Ph1* locus was deleted, expression of *TaZIP4-B2* was also eliminated (*p < 0.05*), but there was no apparent increase in the transcription of the *ZIP4* homologues on homoeologous group 3 chromosomes, to compensate for the absence of *ZIP4* on chromosome 5B (*p > 0.05*) (Fig. 1; Supplementary Table 1). Thus, RNA-seq data revealed that the expression of *ZIP4* was derived mainly from the gene (*TaZIP4-B2*) on chromosome 5B, within the *Ph1* locus.

**Fig. 2:**
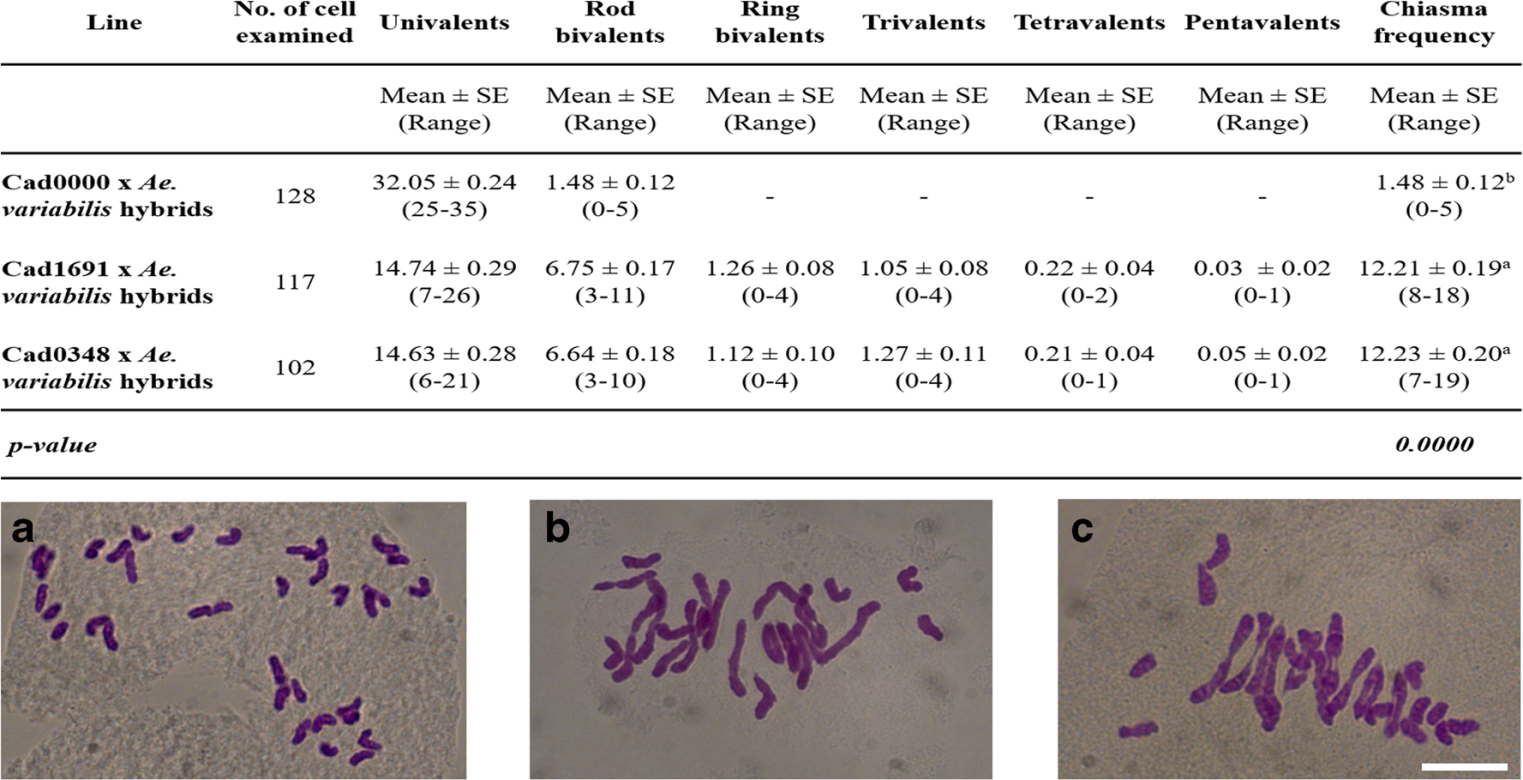
Homoeologous CO frequency at metaphase I is increased in *Tazip4-B2* mutant line-*Ae. variabilis* hybrids (**b**, **c**), in comparison to the wild-type Cadenza-*Ae. variabilis* hybrid (**a**). The number of univalents, bivalents, multivalents and chiasma frequency were scored at meiotic metaphase I in Cadenza0000 (*TaZIP4-B2*) × *Ae. variabilis* hybrids, and in Cadenza1691 (*Tazip4-B2*) × *Ae. variabilis* hybrids and Cadenza0348 (*Tazip4-B2*) × *Ae. variabilis* hybrids. The same letter indicates no differences between *TaZIP4-B2* (**a**) and *Tazip4-B2* hybrids (**b**, **c**) in metaphase I at *P* < 0.05. *Scale bar* represents 10 μm for all panels

### *TaZIP4-B2* suppresses homoeologous COs in wheat-*Ae. variabilis* hybrids

The protein-coding sequences of 1200 EMS mutant lines (Rakszegi et al. 2010) from hexaploid wheat cv. Cadenza have been recently sequenced using exome-capture and displayed to allow the identification of millions of mutations in the sequenced genes using the www.wheat-tilling.com database (Krasileva et al. 2017). The mutations identified are accessible using the wheat survey sequence (Marcussen et al. 2014) via this database, which includes their location within the gene, and the predicted effect that each variant has on its protein. Simply searching the database (www.wheat-tilling.com) reveals those plants possessing mutations in the target genes, as well as a list of all mutations possessed by the plant (Krasileva et al. 2017). We selected seven of the 1200 EMS mutant lines, which possessed potentially interesting mutations within *TaZIP4-B2* (Traes_5BL_9663AB85C.1) (Supplementary Table 2). Five of these mutant lines exhibited regular pairing at meiotic metaphase I, so were not taken further. These lines possessed a missense mutation, which indicates that amino acid changes within ZIP4 in these *Tazip4-B2* lines did not affect its function. However, two of the mutant lines (Cadenza1691 and Cadenza0348) showed reduced number of COs in cytological analysis, suggesting that their *Tazip4-B2* mutations did exhibit a phenotype. Both lines were selected for wide crossing studies with wild relatives to score the effect of their *Tazip4-B2* mutations on homoeologous CO frequency in the wheat *Tazip4-B2* mutant-wild relative hybrids, as compared to non-mutagenised wheat-wild relative hybrids. Null segregants were not available, so wild-type Cadenza lines were used as controls. Mutations within *TaZIP4-B2* were verified by sequencing, and primers were designed to the mutated regions to follow mutated genes during crossing (Supplementary Fig. 3 and Materials and Methods). *Tazip4-B2*, within the Cadenza1691 mutant line, possessed a missense mutation within the Spo22 domain (C to T change leading to an A167V), shown to be important for ZIP4 function (Perry et al.^2005^). *Tazip4-B2*, within the Cadenza0348 mutant line, possessed a nonsense mutation (a premature stop codon: G to A change leading to W612*) (Supplementary Fig. 3). In addition to these *Tazip4-B2* mutations, the two mutant lines also possessed mutations (mostly missense, but also splice or stop codons) within the coding sequences of 106 other shared genes. Sixteen of these genes, including *TaZIP4-B2*, were located on chromosome 5B. However, none of the genes apart from *TaZIP4-B2* were located within the 2.5 MB *Ph1* region defined in our previous study (Griffiths et al. 2006; Al-Kaff et al. 2008).

**Fig. 3:**
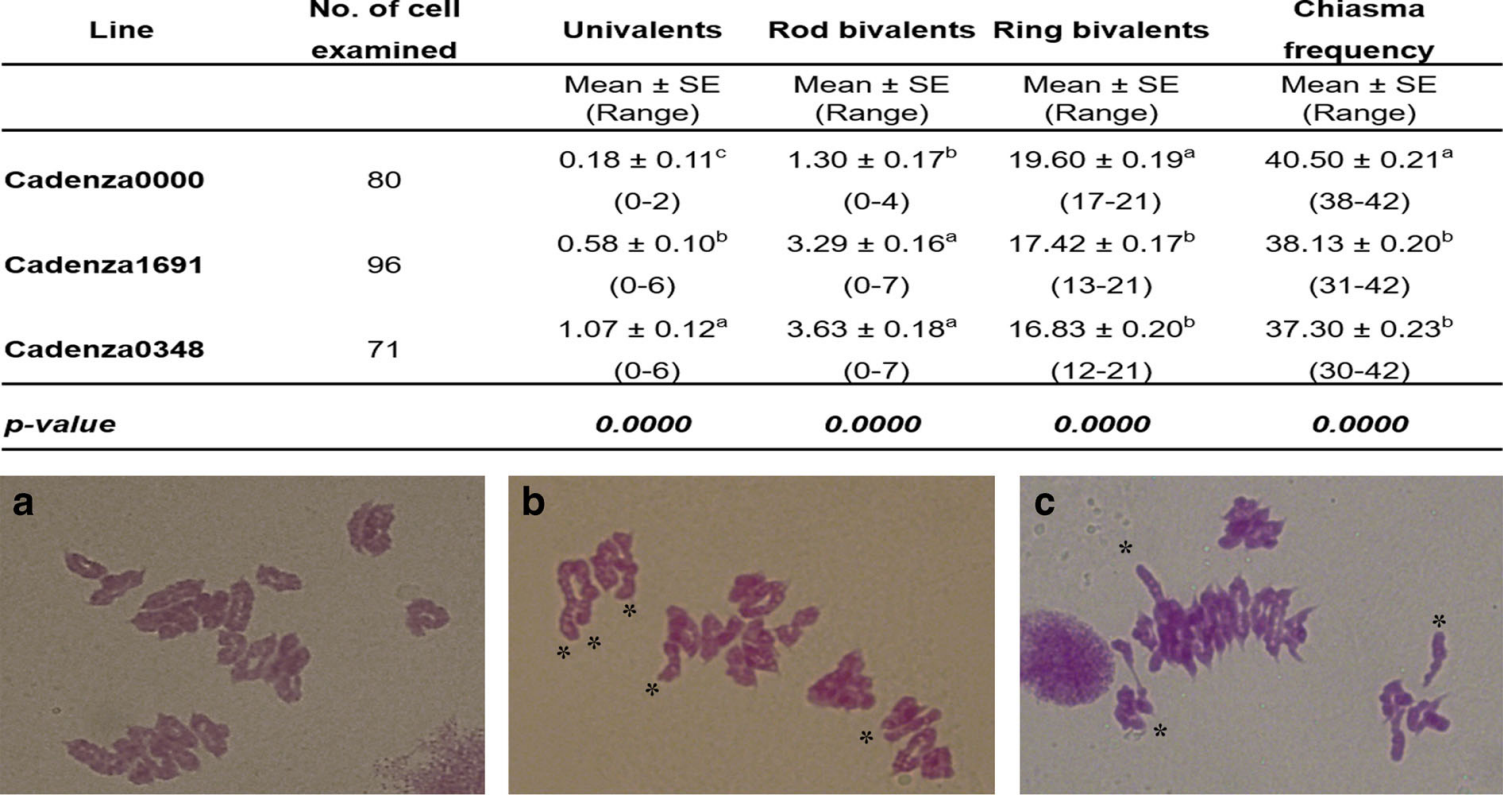
Homologous CO frequency is reduced in *Tazip4-B2* mutant Cadenza lines (**b**, **c**), in comparison to wild-type Cadenza (**a**). The number of univalents, bivalents and chiasma frequency were scored at meiotic metaphase I in Cadenza0000 (*TaZIP4-B2*) and in Cadenza1691 (*Tazip4-B2*) and Cadenza0348 (*Tazip4-B2*). *Asterisks* indicate the presence of rod bivalents in both mutant Cadenza lines. The same letter indicates no differences between *TaZIP4-B2* (**a**) and *Tazip4-B2* wheat (**b**, **c**) in metaphase I at *P* < 0.05. *Scale bar* represents 10 μm for all panels

Compared to the chromosome 5B deletion mutant—wild relative hybrid—no other wheat chromosome deletion mutants have previously been reported as exhibiting a similar level of homoeologous CO formation at metaphase I (Riley and Chapman 1958; Sears 1977). For example, the 3D locus *Ph2* exhibits a four-fold lower level of induction compared to *Ph1* (Prieto et al. 2005). Equally, deletion of regions of chromosome 5B apart from the 2.5 MB *Ph1* region does not result in homoeologous CO formation at metaphase I when the lines are crossed with wild relatives (Roberts et al. 1999; Griffiths et al. 2006; Al-Kaff et al. 2008). Sears (1977) used such crosses between hexaploid wheat cv. Chinese Spring, both in the presence and absence (*ph1b* deletion) of *Ph1*, and the wild relative tetraploid *Aegilops kotschyi* (also termed *Ae. variabilis*), to show that homoeologous COs are induced when the *Ph1* locus is deleted. Interspecific hybrids of the *ph1b* mutant and wild relatives have been subsequently used in plant breeding programmes for introgression purposes (Sears 1977). Sears (1977) observed one rod bivalent at metaphase I in the presence of *Ph1*, and 6.35–7.28 rod bivalents in the *Ph1* absent hybrids. Chiasma frequency in the hexaploid wheat-*Ae. kotschyi* or hexaploid wheat-*Ae. variabilis* hybrids was between 1 and 3 in the presence of *Ph1* and 11–14 in the absence of *Ph1* (Sears 1977; Farooq et al. 1990; Fernández-Calvín and Orellana 1991; Kousaka and Endo 2012).

In this study, both *Tazip4-B2* mutant Cadenza lines, as well as a wild-type Cadenza (*TaZIP4-B2*), were crossed with *Ae. variabilis*. The frequency of univalents, bivalents, multivalents and total chiasma frequency was scored at meiotic metaphase I in the resulting F_1_ hybrid (Fig. 2). In these hybrids, there were similar numbers of rod bivalents to that reported by Sears (1977), with 6.75 (SE 0.17) (Cadenza1691) and 6.64 (SE 0.18) (Cadenza0348) rod bivalents at metaphase I in the *Tazip4-B2* mutants, and 1.48 rod bivalents (SE 0.12) at metaphase I in the wild-type Cadenza. Moreover, the chiasma mean frequency was 1.48 (SE 0.12) in the presence of *Ph1* (*TaZIP4-B2*) and 12.21 (SE 0.19) and 12.23 (SE 0.20) in the Cadenza1691-*Ae. Variabilis* and Cadenza0348-*Ae. variabilis* hybrids, respectively. The observed chiasma frequencies at metaphase I, in the two *Tazip4-B2* mutant line-*Ae. variabilis* hybrids, are similar to those previously reported at metaphase I in the *Ph1* deletion mutant (*ph1b*)-*Ae*. *variabilis* hybrids. Thus, the data indicate that *TaZIP4-B2* within the *Ph1* locus is likely to be involved in the suppression of homoeologous COs.

### *Tazip4-B2* mutant Cadenza lines show no multivalents

The frequencies of meiotic associations at metaphase I in hexaploid wheat and the *Ph1* deletion mutant (*ph1b*) have been reported previously (Martín et al. 2014). Martín et al. (2014) observed 20 ring bivalents and one rod bivalent, with a chiasma frequency of 40.97 in the presence of the *Ph1* locus. However, the number of ring bivalents decreased to 14.83, with a reduced chiasma frequency of 35.78, while the number of univalents, rod bivalents, trivalents and tetravalents increased to 0.80, 4.73, 0.20 and 0.37, respectively, when the *Ph1* locus was absent. The number of univalents, bivalents, multivalents and chiasma frequency at meiotic metaphase I was also scored in both the *Tazip4-B2* mutant lines and in the wild-type Cadenza (Fig. 3). The *Tazip4-B2* mutant lines exhibited a reduction in the number of ring bivalents at metaphase I, and a slight increase in the number of rod bivalents, from a mean of 1.30 (SE 0.17) in the wild-type Cadenza, to 3.29 (SE 0.16) in Cadenza1691 and 3.63 (SE 0.18) in Cadenza0348. This indicates a slight reduction in homologous COs in these *Tazip4-B2* mutant lines. CO frequency was a mean of 40.50 (SE 0.21) in the wild-type Cadenza, 38.13 (SE 0.20) in Cadenza1691 and 37.30 (SE 0.23) in Cadenza0348. These observed chiasma frequencies at metaphase I in the two mutant Cadenza lines are again similar to those previously reported at metaphase I in wheat in the absence of the *Ph1* locus. However, no multivalents were observed, and there was no significant increase in the number of univalents at metaphase I in the *Tazip4-B2* mutant lines, as is normally observed in *Ph1* deletion mutants (Roberts et al. 1999).

If *Tazip4-B2* mutants had enabled homoeologues to synapse while failing to CO, then a significant increase in univalents would be expected, but this was not observed. This suggests that homoeologous synapsis may not be significantly affected by *TaZIP4-B2*. On the other hand, the lack of multivalents at metaphase I suggests that both mutant lines will exhibit a reduced level of homoeologous exchange or chromosome translocation to that observed in the CS *ph1b* mutant. The *ph1b* mutant line has been reported to accumulate extensive background translocations over multiple generations due to homoeologous synapsis and COs (Sánchez-Morán et al. 2001). Thus, the apparent lack of multivalents in the *Tazip4-B2* mutant lines could allow their exploitation for introgression purposes during plant breeding programmes, rather than the current *ph1b* line.

Thus, seven lines carrying mutations within the *TaZIP4-B2* gene were screened for a phenotype with reduced homologous crossover at metaphase I. Of these, two lines were identified with this phenotype, one carrying a nonsense mutation within *TaZip4-B2*, and the other carrying a mutation in one of the key functional domains of *TaZip4-B2.* When crossed with *Ae. variabilis*, both of these lines also exhibited increased homoeologous crossover at metaphase I in the resulting hybrid, suggesting that the two phenotypes were linked. This is consistent with our previous study, which scored for the absence of the *Ph1* locus by the occurrence of reduced homologous crossover in wheat and increased homoeologous crossover in wheat-rye hybrids (Roberts et al. 1999; Al-Kaff et al. 2008). Therefore, lines with increased homoeologous crossover were identified without an initial screen for the desired phenotype. Until now, the only way by which homoeologous crossover at metaphase I could be increased to this extent in wheat-wild relative hybrids was by deletion of the *Ph1* locus, defined as a deletion effect phenotype specific to chromosome 5B. However, an alternative way of reproducing the *Ph1* deletion effect would be to use EMS treatment to generate nonsense or truncation mutations in the homoeologous crossover-suppressing gene within the *Ph1* locus. Analysis of the 1200-line TILLING population revealed that in any given mutant line, 1.5% of genes will have a truncation allele and 2% a missense allele (Krasileva et al. 2017). Thus, the probability of two mutant lines both sharing a truncation or missense mutation by chance in a second gene is *P* < 0.0005. The probability that two mutated genes will be located on the same chromosome is extremely low (2.4 × 10^−5^), and the probability that they will both be located within the *Ph1* region is even lower (2.4 × 10^−7^). Thus, it is extremely unlikely that the increased homoeologous crossover phenotype found in both *Tazip4-B2* mutant lines results from a nonsense mutation in a further gene independently linked with *Tazip4-B2* within the *Ph1* locus.

In terms of follow-on studies based around the observations reported here, we are currently backcrossing (BC) both *Tazip4-B2* lines to clean up background mutations, as well as transferring *Tazip4-B2* into the highly crossable hexaploid wheat cv. Chinese Spring. We are currently at BC_3_. BC lines will be made available once this exercise is complete. We have also complemented the approach of identifying chemically induced *Tazip4-B2* mutants, by exploiting CRISPR to generate a large deletion within *TaZIP4-B2.* Initial analysis reveals that this *Tazip4-B2* line has a similar phenotype to the two *Tazip4-B2* Cadenza lines described above. We are currently segregating the transgenes away. *TaZIP4-B2* is also being over-expressed to assess whether increased ZIP4 levels reduce homologous crossover. Our current hypothesis is that a higher ZIP4 level is optimal for homologous crossover, and a lower ZIP4 level is optimal for homoeologous crossover. Reducing or increasing ZIP4 around the optimal levels reduces the frequency of crossover. Interestingly, increasing the copy number of 5B chromosomes carrying *TaZIP4-B2* reduces homologous crossover (Feldman 1966). In addition, a recent study identified another recombination pathway gene exhibiting dosage-dependent control on crossover (Ziolkowski et al. 2017). Thus, ZIP4 levels optimal for homologous crossover may be too high for homoeologous crossover, while the lower ZIP4 levels optimal for homoeologous crossover are too low for optimum homologous crossover. Finally, we are exploiting deletions of the less complex, homoeologous Cdk/methyl transferase locus on chromosome 5D, to shed further light on the role of these genes in the regulation of chromosome synapsis.

In summary, two *Tazip4-B2* mutants were identified through a non-GM route, which can be exploited as an alternative to the CS *ph1b* mutant. Seeds for both mutants have been deposited with the Germplasm Resource Unit at the John Innes Centre (www.jic.ac.uk/research/germplasm-resources-unit). The accession number of seeds for Cad0348 is W10336 and for Cad1691 is W10337. The seeds for both lines are available on request, free of intellectual property restrictions.

## Electronic supplementary material

ESM 1 (DOCX 38 kb).

Supplementary Table 1 Detailed information of all transcripts obtained by Kallisto and the statistical analysis of transcripts per million (TPM) in wheat chromosomes 3AL, 3B, 3DL and 5BL, both in presence (WT) and in absence (*ph1b* deletion) of the *Ph1* locus. Data represent mean values ± standard error (SE) from RNA samples collected at late leptotene-early zygotene stage in WT and in *ph1b* deletion. (XLSX 13 kb).

Supplementary Table 2 Detailed information on the seven EMS mutant lines selected as possessing potentially interesting mutations within *TaZIP4-B2*(Traes_5BL_9663AB85C.1). The two mutant lines (Cadenza1691 and Cadenza0348) which showed reduced number of COs in Cadenza mutant lines are indicated in bold. (XLSX 15 kb).
