# Supplementary material for: Exploiting the ZIP4 homologue within the wheat Ph1 locus has identified two lines exhibiting homoeologous crossover in wheat-wild relative hybrids
Source: Mol Breed. 2017 Jul 18;37(8):95. doi: 10.1007/s11032-017-0700-2 (PMC5515957; doi:10.1007/s11032-017-0700-2)
Supplement: Supplementary file 1 — (DOCX 38 kb). [file 11032_2017_700_MOESM1_ESM.docx]

**Supplementary Fig. 1** Alignment of coding DNA (**a**) and amino acid sequences (**b**) from *TaZIP4-B2* (TRIAE_CS42_5BL_TGACv1_404600_AA1305800), *TaZIP4-B1* (TRIAE_CS42_3B_TGACv1_225572_AA0809860), *TaZIP4-A1* (TRIAE_CS42_3AL_TGACv1_195180_AA0645950) and *TaZIP4-D1* (TRIAE_CS42_3DL_TGACv1_251716_AA0884100) CS+ refers to wild type and CS- to the *ph1b* mutant.

**A** Coding DNA sequences

5BL_AA1305800.1 ATGACGATCTCCGAGCTCT-----------------------------------------

3B__AA0809860.1 ATGAAGATCTCCGAGCTCTCCCCCGAGTACCACCGCCCGCCGCCGCAGGCCGCCCACCTC

3AL_AA0645950.1 ATGAAGATCTCCGAGCTCTCCCCGGAGTACCACCGCCCGCCGCCGCACGCCGCCCACCTC

3DL_AA0884100.1 ATGATGATCTCCGAGCTCTCCCCGGAGTACCACCGCCCGCCGCCGCACGCCGCGCACCTC

5BL_AA1305800.1 -CTGAGCTCAAGCGGGT---------CGTCGATCTGTACGACGCCCCCGACCCCTCCCCC

3B__AA0809860.1 ACCGACCTCGCGCGGGTCGTCGCGGACGTCGAGCGGTACGACGCCCCCGACCCCTCCCCC

3AL_AA0645950.1 ACCGACCTCGCGCGGGTCGTCGCGGACGTCGAGCGGTACGACGCCCCCGACCCCTCCCCC

3DL_AA0884100.1 ACCGACCTCGCGCGGGTCGTCGCGGACGTCGAGCGGTACGACGCCCCCGACCCCTCCCCC

5BL_AA1305800.1 CCGGAGAAGCTCGTCGCCGACTTCCGCCGCGTCCTCACCAACCTCGGCTCCGCCGCCTCC

3B__AA0809860.1 CCGGAGAAGCTCGCCGCCGACTTCCGCCGCGTGCTCACCAACCTCGGCTCCGCCGCCTCG

3AL_AA0645950.1 CCGGAGAAGCTCGCCGCCGACTTCCGCCGCGTGCTCACCAACCTCGGCTCCGCCGCCTCG

3DL_AA0884100.1 CCGGAGAAGCTCGCCGCCGACTTCCGCCGCGTGCTCACCAACCTCGGCTCCGCCGCCTCG

5BL_AA1305800.1 TCCCTCACCGACGCCTTACCCAGGCTACAGTTGTACAATCTCGGCAACCGCCTCTGGAAA

3B__AA0809860.1 TCCCTCACCGACGCCTG---CAGGCTCCAGATATGGAAGCTCGCCACCCGCCTCTGGAAC

3AL_AA0645950.1 TCCCTCACCGACCCCTG---CAGGCTCCAGATATGGAAGCTCGCCACCCGCCTCTGGAAC

3DL_AA0884100.1 TCCCTCACCGACCCCTG---CAGGCTCCAGATATGGAAGCTCGCCACCCGCCTCTGGAAC

5BL_AA1305800.1 GCCGTGGACAAGCGCGCCAACTCCGCCGCGCTGGCCCTGGGTCCCTCCGCCCGCGCCGCC

3B__AA0809860.1 GCCGTGGTCGACCGCGCCAACTCCGCCGCGCTGGCCCGGGGCCCCTCCGCGCGCGCCGCC

3AL_AA0645950.1 GCCGTCGTCGACCGCGCCAACTCCGCCGCGCTGGCCCGGGGCCCCTCCGCGCGCGCCGCC

3DL_AA0884100.1 GCCGTCGTCGACCGCGCCAACTCCGCCGCGCTGGCCCGGGGCCCCTCCGCGCGCGCCGCC

5BL_AA1305800.1 GAGGCGGAGGTCAGGCAGGTGGCGCCGGAGCTGCTCCTCCTCGCCGGCTTCC--------

3B__AA0809860.1 GAGGCGGAGATCAGGCAGGCGGCGCCGGAGCTGCTCCTCCTCGCCGGCTGCCCCGACGAG

3AL_AA0645950.1 GAGGCGGAGATCAGGCAGGCCGCGCCGGAGCTCCTCCTCCTCGCCGGCTGCCCCGACGAG

3DL_AA0884100.1 GAGGCGGAGATCAGGCAGGCGGCGCCGGAGATCCTCCTCCTCGCCGGCTGCCCCGACGAG

5BL_AA1305800.1 -------------CCGTCAAGGCCGCATCCTTCTTCCTCCGCGCCGGCCAGGGGTGGCTC

3B__AA0809860.1 GTCCCCCTCGCCGCCGCCAAGGCCGCCTCCTTCTTCCTCCGCGCCGGCCAGGAGTGGCTC

3AL_AA0645950.1 GTCCCCCTCGCCGCCGCCAAGGCCGCCTCCTTCTTCCTCCGCGCCGGCCAGGAGTGGCTC

3DL_AA0884100.1 GTCCCCCTCGCCGCCGCCAAGGCCGCCTCCTTCTTCCTCCGCGCCGGCCAGGAGTGGCTC

5BL_AA1305800.1 GACCTCGGCCGCGTCGACCTCGCCACCGCCTGCTTCGACAAGGCCACGCCGCTCGTCTCC

3B__AA0809860.1 GACCTCGGCCGCGTCGACCTCGCCACCGCCTGCTTCGAGAAGGCCACGCCGCTCGTCTCC

3AL_AA0645950.1 GGCCTCGGCCGCGTCGACCTCGCCACCGCCTGCTTCGAGAAGGCCACGCCGCTCGTCTCC

3DL_AA0884100.1 GACCTCGGCCGCGTCGACCTCGCCACCGCCTGCTTCGAGAAGGCCACGCCGCTCGTCTCC

5BL_AA1305800.1 GCGCTCGCCACGGAGGATGAGCGACGCATCCTGCTCGGCCTCAACCTCGCGCGGGCGCGC

3B__AA0809860.1 ACCCCCGCCACGGAGGATGAGCGGGACGTCCTGCTCGGCCTCAACCTCGCGCGGGCGCGC

3AL_AA0645950.1 GCCCCCGCCGCGGAGGAGGAGCGGGACGTCCTGCTCGGCCTCAACCTCGCGCGGGCGCGC

3DL_AA0884100.1 GCCCCCGCCGCGGAGGAGGAGCGGGACGTCCTGCTCGGCCTCAACCTCGCGCGGGCGCGC

5BL_AA1305800.1 GCGGCGTCCGACGCGGGCGATCAGTCCCTCGCCGTCGCGCTGCTCGGCCGCTCCAAGTCC

3B__AA0809860.1 GCCGCGTCCGACGCGGGCGATCAGTCCCTCGCCGTCGCGCTGCTCGGCCGCTCCAA----

3AL_AA0645950.1 GCCGCGTCCGACGCGGGCGATCAGTCCCTCGCCGTCGCGCTGCTCGGTCGCTCCAA----

3DL_AA0884100.1 GCCGCGTCCGACGCGGGCGATCAGTCCCTCGCCGTCGCGCTGCTCGGCCGCTCCAA----

5BL_AA1305800.1 CTCGCGTCCCTCGCGTCCGCGCCCCGCAAGTATACCAGATCCCTCGCCGAGGGGTACCTG

3B__AA0809860.1 -----GCCCCTCGCGTCCGCGTCCCCCAAGGGGACCAGATCCCTCGCCGAGGGCTACCTG

3AL_AA0645950.1 -----GCCCCTCGCGTCCGCCTCCCCCAAGGGGACCAGATCCCTCGCCGAGGGCTACCTG

3DL_AA0884100.1 -----GCCCCTCGCGTCCGCGTCCCCCAAGGGGACCAGATCCCTCGCCGAGGGCTACCTG

5BL_AA1305800.1 TACGTCGGCGAGGCCACGCTCTTCGCGAAGCCCTCCGACCCCGCCGTCGTAGCGTCCATG

3B__AA0809860.1 TCCGTCGGCGAGGCCGCGCTATCCACCAAACCTTCTGACCCCGCCGTTGGAGCCTCCAGC

3AL_AA0645950.1 TCCGTCGGCGAGGCCGCGCTATCCACCAAACCCTCTGACCCCGCCGTTGGAGCGTCCAGC

3DL_AA0884100.1 TCCGTCGGCGAGGCCGCGCTCTCCGCGAAACCCTCTGATCCCGCCGTTGGAGCGTCCAGC

5BL_AA1305800.1 CTCCTCACCGAGGCGCTAGATCTCTTCCAGAAGCTGCCCCGCCCCCCAAAAAAAAAGAAG

3B__AA0809860.1 CTCATCACCGAGGCGCTGGATCTCTTC---------------------------GAGAAG

3AL_AA0645950.1 CTCATCACCGAGGCGCTAGATCTCTTC---------------------------GAGAAG

3DL_AA0884100.1 CTCATCACCGAGGCGCTAGATCTCTTC---------------------------GAGAAG

5BL_AA1305800.1 CTGGCCTCACCCTCCCCCTCCAGCTC------------------------GGAGCAAAAG

3B__AA0809860.1 CTGGCCTCCCCCTCCCCCTCCAGCGCCAGTCCCAAGACCCCAAATCTCCAGGAGCAAAAG

3AL_AA0645950.1 CTGGCCTCCCCCTGCCCCTCCAGCGCCAGTCCCAAGACCCCAAATCTCCAGCAGCAAAAG

3DL_AA0884100.1 CTGCCCTCCCCCTCCCCCTCCAGCGCCAGTCCCAAGACCCCGAATCTCCAGCAGCAAAAG

5BL_AA1305800.1 GGTCGATGCCTCCGCTACCTTGCCCTCGAGCATCACAAAGCCAAGGAACACGCTGACGTC

3B__AA0809860.1 GGTCGATGCCTCCGCTACCTTGCCCTCGAGCGTCACCAAGCCAAGGATTACGAGGGCGTC

3AL_AA0645950.1 GGTCGATGCCTCCGCTACCTTGCCCTGGAGCGTCTCGAAGCCAAGGACCACGAGGGCGTC

3DL_AA0884100.1 GGTCGATGCCTCCGCTACCTTGCCCTCGAGCGTCTCGAAGCCAAGGACCACGAGGGCGTC

5BL_AA1305800.1 CTGTTTTTTATCCATGCCTCGAGGGATTTAATTGGGGAGGCTGATGAGCACCCGAGCATG

3B__AA0809860.1 CTGCGTTGTGTCCAGGTCTCGAGGGCTTCAGTGGGGCAGGCGGATGAGCACGCGAGCGTG

3AL_AA0645950.1 CTGCGTTGTGTCCAGGTCTCGAGGGCTTCAGTGGGGCAGGCGGACGAGCACCCGAGCATG

3DL_AA0884100.1 CTGCGTTGTGTCCAGGTCTCCAGGGCTTCAGTGGGGCAGGCGAACGAGCACCCGAGCATG

5BL_AA1305800.1 GGGTTCATGGCCCTGCACGCGTGGCTTGGTACCGGCAACTTGGCGGAGGCCGAGAGGGAG

3B__AA0809860.1 GGGTTCATGGCGCTGCACGCGTGGCTTGGTACCGGGAACTTGCCGGAGGCCGAGAGGGAG

3AL_AA0645950.1 GGGTTCATGGCCCTGCACGCGTGGCTTGGCACCGGGAACTTGGCGGAGGCCGAGAGGGAG

3DL_AA0884100.1 GGGTTCATGGCCCTGCACGCGTGGCTTGGTACCGGGAACTTGGCGGAGGCCGAGAGGGAG

5BL_AA1305800.1 CTCGAGAGACTCATGGCCAACGCCAACGCATCAGAGAGTCTATGCGTGGGGGCTGCTGAG

3B__AA0809860.1 CTCGAGAGACTCATGGCCAACGCCAACGCATCAGAGAGTCTATGCGTGGGGGCTGCTGAG

3AL_AA0645950.1 CTCAAGAGAATCATGGCCAACGCCAACGCACCAGAGAATGTATGCGTGGGGGCAGCTGAG

3DL_AA0884100.1 CTCGAGAGAATCATGGCCAACGCCAACGCACCAGAGAATGTATGCGTGGGGGCAGCTGAG

5BL_AA1305800.1 GTGTACCTTGCCTCCGCGGGGCCTGAGGCTGCACGCAAGGTGCTCGTTGCACTTGTTGCA

3B__AA0809860.1 GTGTACCTTGCCTCCGCGGGGCCTGAGGCTGCACGCAAGGTGCTCGTTGCACTTGTTGCA

3AL_AA0645950.1 GTGTACCTTGCCTCTGCGGGGCCTGAGGCTGCACGCAAGGTGCTCGTTGCGCTTGCTGCA

3DL_AA0884100.1 GTGTACCTTGCCTCCGCGGGGTCTGAGGCTGCACGCAAGGTGCTCGTTGCGCTTGCTGCA

5BL_AA1305800.1 CGTTGCTGCGCGGGAGTTGCAGCTGCTGCTGTGAGAGTGGTGACCAAGGTAGTTGATTGT

3B__AA0809860.1 CGTTGCCGCGCAGGAGGTGCAGCTGCTGCCGTGAGAGTGGTGACCAAGGTAGTTGATTGT

3AL_AA0645950.1 CGTTGCCGCGCAGGAGGTGCAGCTGCTGCTGTGAGAGTGGTGACCTCGGTGGTTGATGGT

3DL_AA0884100.1 CGTTGCCGCGCAGGAGGTGCAGCTGCTGCTGTGAGAGTGGTGACCACGGTGGTTGATGGT

5BL_AA1305800.1 GGTATCGGTAGCCCTGGGCGCGCAAGGGCGATCGGTGAGCTCGTGTCGGATGAGAGGGTG

3B__AA0809860.1 GGTATCGGTAGCCCTGGGCGCGCAAGGGCGATCGGTGAGCTCGTGTCGGATGAGAGGGTG

3AL_AA0645950.1 GGTATCGGTAGCCCTGGGCGCGCAAGAGCGATCGGTGAGCTCGTGTCGGATGAGAGGGTG

3DL_AA0884100.1 GGTATCGGTAGCCCTGGGCGCGCAAGGGCAATCGGTGAGCTCGTGTCAGATGAGAGGGTG

5BL_AA1305800.1 GTCGCACTGTTCGATGGCCCGGCTAACACCAGTCACCGTGGAGCAATGCATACACTGCTC

3B__AA0809860.1 GTCGCACTGTTCGATGGCCCGGCTAACACCAGTCACCGTGGAGCAATGCATACACTGCTC

3AL_AA0645950.1 GTCGCCCTGTTCGATGGCCCGGCTAACACCAGTCACCGTGGAGCAATGCATACACTGCTC

3DL_AA0884100.1 GTCGCACTGTTCGATGGCCCGGCCAACACCAGTCACCGTAGAGCAATGCATACACTGCTC

5BL_AA1305800.1 TGGAACTGCGGGTTTGAGCATTTCAATGCAAAGAACTACGATATATGTGCGGACTTGTTT

3B__AA0809860.1 TGGAACTGCGGGTTTGAGCATTTCAATGCAAAGAACTACGATATATGTGCGGACTTGTTT

3AL_AA0645950.1 TGGAACTGCGGGTTTGAGCATTTCAATGCAAAGAACTACGATACATGCGCGGACTTGTTT

3DL_AA0884100.1 TGGAACTGCGGGTTTGAGCATTTCAATGCTAAGAACTACGATATATGTGCGGACTTGTTT

5BL_AA1305800.1 GAGACATCGATGCTTTATCTATCCCGTGAAGAGGGAAGCAGAGTCCGGCGAGCACAGTGC

3B__AA0809860.1 GAGACATCGATGCTTTATCTATCCCGTGAAGAGGGAAGCAGAGTCCGGCGAGCACAGTGC

3AL_AA0645950.1 GAGACATCGATGCTTTATCTATCCCGTGAAGAGGGAAGCAGAGCCCGGCGAGCACAGTGC

3DL_AA0884100.1 GAGACATCGATGCTTTATCTATCCCGTGAAGACGGAAGCAGAGTCCGGCGAGCACAGTGC

5BL_AA1305800.1 CTCCGAGTCCTTGCCGTTTGCTATCTAGCCCTTCAACGTCTGGATCGAGCACATGAGTTT

3B__AA0809860.1 CTCCGAGTCCTTGCCGTTTGCTATCTAGCCCTTCAACGTCTGGATCGAGCACATGAGTTT

3AL_AA0645950.1 CTCCGAGTCCTTGCCGTTTGCTATCTAGCCCTTCAACGTCTGGATCGAGCACATGAGTTT

3DL_AA0884100.1 CTCCGAGTCCTTGCCGTTTGCTATCTAGCCCTTAAACGTCTGGATCGAGCACATGAGTTT

5BL_AA1305800.1 GTCAATGAGGCTGACAAGGTGGAACACAATGCCCACTGTGCCTTTATGAAGATCAAAATT

3B__AA0809860.1 GTCGATGAGGCTGACAAGGTGGAACACAATGCCCACTGTGCCTTTATGAAGATCAAAATT

3AL_AA0645950.1 GTCAATGAGGCTGACAAGGTGGAACACAATGCCCATTGTGCCTTTATGAAGATCAAAATT

3DL_AA0884100.1 GTCAATGAGGCTGACAAGGTGGAACACAATGCCCACTGTGCCTTTATGAAGATCAAAATT

5BL_AA1305800.1 CATCTTCAGAAGAACGACGAGGACGAGGCTATCAAGCAGATAAAAACCATGATGGGTTGC

3B__AA0809860.1 CATCTTCAGAAGAACGACGAGGACGAGGCTATCAAGCAGATAAAAACCATGATGGGTTGC

3AL_AA0645950.1 CATCTTCAGAAGAACAACGAGGACGAGGCTATCAAGCAGATAAAAACCATGATGGGTTGC

3DL_AA0884100.1 CATCTTCAGAAGAACGACGAGGACGAGGCTATCAAGCAGATAAAAACCATGATGGGTTGC

5BL_AA1305800.1 ATTGACTTCAATCCCGTGTTCCTAATGCTCACAACTCATGAGGCTATTGCCTGCAAGGCT

3B__AA0809860.1 ATTGACTTCAATCCCGCGTTCCTAATGCTCACAACTCATGAGGCTATTGCCTGCAAGGCT

3AL_AA0645950.1 ATTGACTTCAATCCCACATTCCTAATGCTCACAACTCATGAGGCTATTGCCTGCAAGGCT

3DL_AA0884100.1 ATTGACTTCAATCCCTCGTCCCTAATGCTCACAACTCATGAGGCTATTGCCTGCAAGGCT

5BL_AA1305800.1 GTCCGGGTAGCAGTTGCTTCATTGACCTTCCTTCTTGGTCTCTATTCTGCCGGAAAGCCT

3B__AA0809860.1 GTCCGGGTAGCAGTTGCTTCATTGACCTTCCTTCTTGGTCTCTATTCTGCCGGAAAGCCT

3AL_AA0645950.1 GTCCGGGTAGCAGTTGCTTCATTGACCTTCCTTCTTGGTCTCTATTCTGCCGGAAAGCCT

3DL_AA0884100.1 GTCCGGGTAGCAGTTGCTTCATTGACCTTCCTTCTTGGTCTCTATTCTCCCGGAAAGCCT

5BL_AA1305800.1 ATGCCAGAGCGTGAGGTTACTGTCCTCCGCACTCTTATTGAGCTCCTCCGTCGTGAGCAA

3B__AA0809860.1 ATGCCAGAGCGTGAGGTTACTGTCCTCCGCACTCTTATTGAGCTCCTCCGTCGTGAGCAA

3AL_AA0645950.1 ATGCCAGAGCGTGAGGTTACTGTCCTCCGCACTCTTATTGAGCTCCTCCGTCGTGAGCAA

3DL_AA0884100.1 ATGCCAGAGCGTGAGGTTACTGTCCTCCGCACTCTTATTGAGCTCCTCCGTCGTGAGCAA

5BL_AA1305800.1 GGTACTGAGGATGAGATCCTTAAGTACTCGAGACGTGCCAAGCTACGGATGTCTAACCTT

3B__AA0809860.1 GGTACTGAGGATGAGATCCTGAAGTACTTGAGACGTGCCAAGCTACGGATGTCTGACCTT

3AL_AA0645950.1 GGTACTGAGGATGAGATCCTGAAGTACTCGAGACGTGCCAAGCTACGGATGTCTGACCTT

3DL_AA0884100.1 GGTACTGAGGATGAGATTCTGAAGTACTCGAGACGTGCCAAGCTACGGATGTCTGACCTT

5BL_AA1305800.1 GGCGTGGAAGGCTTTTTTGGCAATGGACCTGTTGGGGCGCGTGAACTGAACTGGTTTGCA

3B__AA0809860.1 GGCGTGGAAGGCTTTTTTGGCAATGGACCTGTTGGGGCGCGTGAACTGAACTGGTTTGCA

3AL_AA0645950.1 GGCGCGGAAGGCTTCTTTGGCAATGGACCTGGTGGGGCGCGTGAACTGAACTGGTTTGCC

3DL_AA0884100.1 GGCGTGGAAGACTTTTTCGGCAATGGACCTGTTGGGACGCGTGAACTGAACTGGTTTGCA

5BL_AA1305800.1 GGCAATAGCTGGAATATGGGTAGAAGGGTGGCTAAGGAGCAAAAGTATGATCTTAGTGCT

3B__AA0809860.1 GGCAATTGCTGGAATATGGGTAGAAGGGTGGCAAAGGAGCAAAAGTATGATCTTAGTGCT

3AL_AA0645950.1 AGCAATAGCTGGAATATGGGTAGAAAGGTGGCAAAGGAGCAGAAGTATGATCTTAGTGCT

3DL_AA0884100.1 GGCAATTGCTGGAATATGGGTAGAAGGGTGGCAAAGGAGCAGAAGTATGATCTTAGTGCT

5BL_AA1305800.1 GAGTTCTTTGAGCTTGCAGCAGAGTTCTTTGGTGGTGCAAGTAATGATGAGGGTGATGGA

3B__AA0809860.1 GAGTTCTTTGAGCTTGCAGCAGAGTTCTTTGGTGGTGCAAGTAATGATGAGGGTGATGGA

3AL_AA0645950.1 GAGTTCTTTGAGCTTGCAGCAGAGTTCTTTGGTGCTGCAAGTAATGATGAGGGTGATGGA

3DL_AA0884100.1 GAGTTCTTTGAGCTTGCAGCAGAGTTCTTTGGTGGTGCAAGTAATGATGAGGGTGATGGA

5BL_AA1305800.1 AATCACCCCACACTTTGCAAAGCGTTAATCATGAGTGTCACTTCCATGCTTAAAGCTGAG

3B__AA0809860.1 AATCACCCCACACTTTGCAAAGCGTTAATCATGAGTGTCACTTCCATGCTTAAAGCTGAG

3AL_AA0645950.1 AATCGCCCCACACTTTGCAAAGCGTTAATCATGAGTGTCACTTCCATGCTTGAAGCTGAG

3DL_AA0884100.1 AATCGCCCCACACTTTGCAAAGCGTTAATCATGAGTGTCACTTCCATGCTTCAAGCTGAG

5BL_AA1305800.1 GAGCTAAACAATTCTCCCTTGTTGGATTCTGATGTTAAAAAAGGTGTTGAGATGCTCAGC

3B__AA0809860.1 GAGCTAAACAATTCTCCCTTGTTGGATTCTGATGTTAAAAAAGGTGTTGAGATGCTCAGC

3AL_AA0645950.1 GAGCTAAACAATTCTCCCTTGTCGGATTCTGATGTTAAAAAAGGTGTTGACATGCTCAGC

3DL_AA0884100.1 GAGCTAAACAATTCTCCCTTGTCGGATTCTGATGTTAAAAAAGGTGTTGAGATGCTCAGC

5BL_AA1305800.1 AGAGCTGGCAAGCTATTACCTTCAATCTGGCCCTCGGGTTCAGTCGCCTCTGATCAGGCT

3B__AA0809860.1 AGAGCTGGCAAGCTATTACCTTCAATCTGGCCCTCGGGTTCAGTCGCCTCTGATCAGGCT

3AL_AA0645950.1 AGAGCTGGCAAGCTATTACCTTCAATCTGGCCCTCGGGTTCAGTTGCCTCTGATCAGGCT

3DL_AA0884100.1 AGAGCTGGCAAGCTATTACCTTCAATCTGGCCCTCGGTTTCAGTCGCCTCTGATCAGGCT

5BL_AA1305800.1 GAGGCCAACATCTTTCTGTTTCTTCATACCTTCTACTCCTACCAACTCCTTGACAGGATG

3B__AA0809860.1 GAGGCCAACATCTTTCTGTTTCTGCATACCTTCTACTCCTACCAACTCCTTGACAGGATG

3AL_AA0645950.1 GAGGCCAATGACTTTCTGTTTCTTCATACCTTCTACTCCTACCAACTCCTTGACAGGATG

3DL_AA0884100.1 GAGGCCAACAACTTTATGTTTCTTCATACCTTCTACTCCTACCAACTCCTTGACAGGATG

5BL_AA1305800.1 GACACCAGCGCACATCCTCAGCAGCTCCAACTAGTCAAGAACTTCGCTTCCTCTAAAGCA

3B__AA0809860.1 GACACCAGCGCACATCCTCAGCAGCTCCAACTAGTCAAGAACTTCGCTTCCTCTAAAGCA

3AL_AA0645950.1 GACACCAGCGCACATCCTCAGCAGCTCCAGCTAGTCAAGAACTTCGCTTCCTCTAAAGCA

3DL_AA0884100.1 GACACCAGCGCACATCCTCAGCAGCTCCAACTAGTCAAGAACTTTGCTTCCTCTAAAGCA

5BL_AA1305800.1 TGCACGCCGTCCCATCTTCTCGTACTTGGGAAAGCTGCTTCTGAAGGCACCCCACCAAAC

3B__AA0809860.1 TGCACGCCGTCCCATCTTCTCGTACTTGGGAAAGCTGCTTCTGAAGGCACCCCACCAAAC

3AL_AA0645950.1 TGCACGCCGTCCCATCTTCTCAAACTTGGAAAAACTGCTTCTGAAGGCACCCCACCAAAC

3DL_AA0884100.1 TGCACGCCGTCCCATCTTCTCAAACTTGGAAAAGCTGCTTCTGAAGGCACCCCACCAAAC

5BL_AA1305800.1 CTGCTGGTTGCTGAATTTTCACTGAAGGCTAGCATCAAGACCGCCCTTGCTTCTCACTCC

3B__AA0809860.1 CTGCTGGTTGCTGAATTTTCACTGAAGGCTAGCATCAAGACCGCCCTTGCTTCTCACTCC

3AL_AA0645950.1 CTGCTGGTTGCTGAATTTTCCCTGAAGGCTAGCATCAAGACCGCCCTTGCTTCTCACTCC

3DL_AA0884100.1 CTTCTAGTTGCTGAATTTTCCCTGAAGGCTAGCATCAAGACCGCCCTTGCTTCTCACTCC

5BL_AA1305800.1 CCAAACTACAGGGTAATCAGTGCTGCCCTCAGGAACCTAGCCTGCCTTGCTGGCTTGCAA

3B__AA0809860.1 CCAAACTACAGGGTAATCAGTGCTGCCCTCAGGAACCTAGCCTGCCTTGCTGGCTTGCAA

3AL_AA0645950.1 CCAAACTACAGGGTAATCAGTGCTGCCCTCAGGAACCTAGCCTGCCTTGCTGGCTTGCAA

3DL_AA0884100.1 CCAAACTACAGGGTAATCAGTGCTGCCCTCAGGAACCTAGCCTGCCTTGCTGGCTTGCAA

5BL_AA1305800.1 GACTTAAGTGGTAGCAAGAGTGATGCAGTGTATGGTGTATATCGACAAGCTTACCAGATC

3B__AA0809860.1 GACTTAAGTGGTAGCAAGAGTGATGCAGTGTATGATGTATATCGACAAGCCTACCAGATC

3AL_AA0645950.1 GACTTAAGTGGTAGCAAGAGTGATGCAGTGTATGATGTATATCGACAAGCCTACCAGATC

3DL_AA0884100.1 GACTTAAGTGGTAGCAAGAGTGATGCAGTGTATGATGTATATCGACAAGCCTACCAGATC

5BL_AA1305800.1 GTGGTTGGACTGAGAGAGGGCGAATATCCATGTGAGGAAGGGCAGTGGCTTGCAGTGTCT

3B__AA0809860.1 GTGGTTGGACTGAGAGAGGGCGAATATCCATGTGAGGAAGGGCAGTGGCTTGCAGTGTCT

3AL_AA0645950.1 TTGGTTGGACTGAGAGATGGTGAATATCCATGTGAGGAAGGGCAGTGGCTTGCAGTGACT

3DL_AA0884100.1 ATGGTTGGACTGAGAGATGGTGAATATCCATGTGAGGAAGGGCAGTGGCTTGCAGTGACT

5BL_AA1305800.1 GCCTGGAACAAGTCATATTTGGCTAGGCGGCTTAATCAAGCTTCAGTTGGTATAAAATGG

3B__AA0809860.1 GCTTGGAACAAGTCATATTTGGCCAGGCGGCTTAATCAAGCTTCCGTTGGTATAAAATGG

3AL_AA0645950.1 GCTTGGAACAAGTCATATTTGGCTAGGCGGCTTAATCAAGCTTCAGTTGGTATAAAATGG

3DL_AA0884100.1 GCCTGGAACAAGTCATATTTGGCTAGGCGGCTTAATCAAGCTTCAGTTGGTATAAAATGG

5BL_AA1305800.1 ATGAAGATGGGATTAGATCTCTCTCGGCATGTTGAGAGCATGAAGAAGTATATAGCAGAC

3B__AA0809860.1 ATGAAGATGGGATTAGATCTCTCTCGGCATGTTGAGAGCATGAAGAAGTATACAGCAGAC

3AL_AA0645950.1 ATGAAGATGGGATTAGATCTCTCTTGGCATGTTGAGAGCATGAAGCAGTATACAGCAGAC

3DL_AA0884100.1 ATGAAGATGGGATTAGATCTCTCTCGGCATGTTGAGAGCATGAAGCAGTATACAGCAGAC

5BL_AA1305800.1 ATGGAGCAATGCCTTGAGTACTTCCAGAAAATGATTCATAGTGAAGCTGGTGAACATGCT

3B__AA0809860.1 ATGGAGCAATGCCTTGAGTACTTCCAGAAAATGATTCATAGTGAAGCTGGTGAACATGCT

3AL_AA0645950.1 ATGGAGCAATGCCTTGAGTACTTCCAGAAATTGTTTCATAGTGAAGCTGGTGAACATGCT

3DL_AA0884100.1 ATGGAGCAATGCCTTGAGTACTTCCAGAAATTGTTTCATAGTGAAGCTGGTGAACATGCT

5BL_AA1305800.1 TTGCTCGGAAAAAACCCTGATGAAGGTAGTCGGCAAGAGGGGGCACCAAGCACAAGT-A-

3B__AA0809860.1 TTGCTCGGAAAAAACCCTGATGAATGTAGTCAGCAAGAGGGGGCACCAAGCACAAGG-AC

3AL_AA0645950.1 TTGCTCGGGAAAAACCCTGATGAATGTAGTCAGCAAGAGGGGGCACCAAGCACAAGT-A-

3DL_AA0884100.1 TTGCTCGGAAAAAACCCTGATGAATGTAGTCAGCAAGAGGGGGCACCAAGCACAAGAGA-

5BL_AA1305800.1 ------TGTCTGGTAGCATGTCTCAGCCTGTCTTAGTATAG

3B__AA0809860.1 CAGGTTCATATGGTGCTGTAC----GGCCATCGCCAGCTAG

3AL_AA0645950.1 ------TGTCTGGTAGCATGTCTCAGCCTGTCCTAGTATAG

3DL_AA0884100.1 ------GATCTGA----------------------------

**B** Amino acid sequences

5BL_AA1305800.1 -----------------MTISELSELKRVVDLYDAPDPSPPEKLVADFRRVLTNLGSAAS

3B__AA0809860.1 MKISELSPEYHRPPPQAAHLTDLARVVADVERYDAPDPSPPEKLAADFRRVLTNLGSAAS

3AL_AA0645950.1 MKISELSPEYHRPPPHAAHLTDLARVVADVERYDAPDPSPPEKLAADFRRVLTNLGSAAS

3DL_AA0884100.1 MMISELSPEYHRPPPHAAHLTDLARVVADVERYDAPDPSPPEKLAADFRRVLTNLGSAAS

5BL_AA1305800.1 SLTDALPRLQLYNLGNRLWKAVDKRANSAALALGPSARAAEAEVRQVAPELLLLAGFPV-

3B__AA0809860.1 SLTDA-CRLQIWKLATRLWNAVVDRANSAALARGPSARAAEAEIRQAAPELLLLAGCPDE

3AL_AA0645950.1 SLTDP-CRLQIWKLATRLWNAVVDRANSAALARGPSARAAEAEIRQAAPELLLLAGCPDE

3DL_AA0884100.1 SLTDP-CRLQIWKLATRLWNAVVDRANSAALARGPSARAAEAEIRQAAPEILLLAGCPDE

5BL_AA1305800.1 ------KAASFFLRAGQGWLDLGRVDLATACFDKATPLVSALATEDERRILLGLNLARAR

3B__AA0809860.1 VPLAAAKAASFFLRAGQEWLDLGRVDLATACFEKATPLVSTPATEDERDVLLGLNLARAR

3AL_AA0645950.1 VPLAAAKAASFFLRAGQEWLGLGRVDLATACFEKATPLVSAPAAEEERDVLLGLNLARAR

3DL_AA0884100.1 VPLAAAKAASFFLRAGQEWLDLGRVDLATACFEKATPLVSAPAAEEERDVLLGLNLARAR

5BL_AA1305800.1 AASDAGDQSLAVALLGRSKSLASLASAPRKYTRSLAEGYLYVGEATLFAKPSDPAVVASM

3B__AA0809860.1 AASDAGDQSLAVALLGRSKPLAS---ASPKGTRSLAEGYLSVGEAALSTKPSDPAVGASS

3AL_AA0645950.1 AASDAGDQSLAVALLGRSKPLAS---ASPKGTRSLAEGYLSVGEAALSTKPSDPAVGASS

3DL_AA0884100.1 AASDAGDQSLAVALLGRSKPLAS---ASPKGTRSLAEGYLSVGEAALSAKPSDPAVGASS

5BL_AA1305800.1 LLTEALDLFQKLPRPPKKKKLASPSPSSSEQKGRCLRYLALEHHKAKEHADVLFFIHASR

3B__AA0809860.1 LITEALDLFEKLASPSPSS-ASPKTPNLQEQKGRCLRYLALERHQAKDYEGVLRCVQVSR

3AL_AA0645950.1 LITEALDLFEKLASPCPSS-ASPKTPNLQQQKGRCLRYLALERLEAKDHEGVLRCVQVSR

3DL_AA0884100.1 LITEALDLFEKLPSPSPSS-ASPKTPNLQQQKGRCLRYLALERLEAKDHEGVLRCVQVSR

5BL_AA1305800.1 DLIGEADEHPSMGFMALHAWLGTGNLAEAERELERLMANANASESLCVGAAEVYLASAGP

3B__AA0809860.1 ASVGQADEHASVGFMALHAWLGTGNLPEAERELERLMANANASESLCVGAAEVYLASAGP

3AL_AA0645950.1 ASVGQADEHPSMGFMALHAWLGTGNLAEAERELKRIMANANAPENVCVGAAEVYLASAGP

3DL_AA0884100.1 ASVGQANEHPSMGFMALHAWLGTGNLAEAERELERIMANANAPENVCVGAAEVYLASAGS

5BL_AA1305800.1 EAARKVLVALVARCCAGVAAAAVRVVTKVVDCGIGSPGRARAIGELVSDERVVALFDGPA

3B__AA0809860.1 EAARKVLVALVARCRAGGAAAAVRVVTKVVDCGIGSPGRARAIGELVSDERVVALFDGPA

3AL_AA0645950.1 EAARKVLVALAARCRAGGAAAAVRVVTSVVDGGIGSPGRARAIGELVSDERVVALFDGPA

3DL_AA0884100.1 EAARKVLVALAARCRAGGAAAAVRVVTTVVDGGIGSPGRARAIGELVSDERVVALFDGPA

5BL_AA1305800.1 NTSHRGAMHTLLWNCGFEHFNAKNYDICADLFETSMLYLSREEGSRVRRAQCLRVLAVCY

3B__AA0809860.1 NTSHRGAMHTLLWNCGFEHFNAKNYDICADLFETSMLYLSREEGSRVRRAQCLRVLAVCY

3AL_AA0645950.1 NTSHRGAMHTLLWNCGFEHFNAKNYDTCADLFETSMLYLSREEGSRARRAQCLRVLAVCY

3DL_AA0884100.1 NTSHRRAMHTLLWNCGFEHFNAKNYDICADLFETSMLYLSREDGSRVRRAQCLRVLAVCY

5BL_AA1305800.1 LALQRLDRAHEFVNEADKVEHNAHCAFMKIKIHLQKNDEDEAIKQIKTMMGCIDFNPVFL

3B__AA0809860.1 LALQRLDRAHEFVDEADKVEHNAHCAFMKIKIHLQKNDEDEAIKQIKTMMGCIDFNPAFL

3AL_AA0645950.1 LALQRLDRAHEFVNEADKVEHNAHCAFMKIKIHLQKNNEDEAIKQIKTMMGCIDFNPTFL

3DL_AA0884100.1 LALKRLDRAHEFVNEADKVEHNAHCAFMKIKIHLQKNDEDEAIKQIKTMMGCIDFNPSSL

5BL_AA1305800.1 MLTTHEAIACKAVRVAVASLTFLLGLYSAGKPMPEREVTVLRTLIELLRREQGTEDEILK

3B__AA0809860.1 MLTTHEAIACKAVRVAVASLTFLLGLYSAGKPMPEREVTVLRTLIELLRREQGTEDEILK

3AL_AA0645950.1 MLTTHEAIACKAVRVAVASLTFLLGLYSAGKPMPEREVTVLRTLIELLRREQGTEDEILK

3DL_AA0884100.1 MLTTHEAIACKAVRVAVASLTFLLGLYSPGKPMPEREVTVLRTLIELLRREQGTEDEILK

5BL_AA1305800.1 YSRRAKLRMSNLGVEGFFGNGPVGARELNWFAGNSWNMGRRVAKEQKYDLSAEFFELAAE

3B__AA0809860.1 YLRRAKLRMSDLGVEGFFGNGPVGARELNWFAGNCWNMGRRVAKEQKYDLSAEFFELAAE

3AL_AA0645950.1 YSRRAKLRMSDLGAEGFFGNGPGGARELNWFASNSWNMGRKVAKEQKYDLSAEFFELAAE

3DL_AA0884100.1 YSRRAKLRMSDLGVEDFFGNGPVGTRELNWFAGNCWNMGRRVAKEQKYDLSAEFFELAAE

5BL_AA1305800.1 FFGGASNDEGDGNHPTLCKALIMSVTSMLKAEELNNSPLLDSDVKKGVEMLSRAGKLLPS

3B__AA0809860.1 FFGGASNDEGDGNHPTLCKALIMSVTSMLKAEELNNSPLLDSDVKKGVEMLSRAGKLLPS

3AL_AA0645950.1 FFGAASNDEGDGNRPTLCKALIMSVTSMLEAEELNNSPLSDSDVKKGVDMLSRAGKLLPS

3DL_AA0884100.1 FFGGASNDEGDGNRPTLCKALIMSVTSMLQAEELNNSPLSDSDVKKGVEMLSRAGKLLPS

5BL_AA1305800.1 IWPSGSVASDQAEANIFLFLHTFYSYQLLDRMDTSAHPQQLQLVKNFASSKACTPSHLLV

3B__AA0809860.1 IWPSGSVASDQAEANIFLFLHTFYSYQLLDRMDTSAHPQQLQLVKNFASSKACTPSHLLV

3AL_AA0645950.1 IWPSGSVASDQAEANDFLFLHTFYSYQLLDRMDTSAHPQQLQLVKNFASSKACTPSHLLK

3DL_AA0884100.1 IWPSVSVASDQAEANNFMFLHTFYSYQLLDRMDTSAHPQQLQLVKNFASSKACTPSHLLK

5BL_AA1305800.1 LGKAASEGTPPNLLVAEFSLKASIKTALASHSPNYRVISAALRNLACLAGLQDLSGSKSD

3B__AA0809860.1 LGKAASEGTPPNLLVAEFSLKASIKTALASHSPNYRVISAALRNLACLAGLQDLSGSKSD

3AL_AA0645950.1 LGKTASEGTPPNLLVAEFSLKASIKTALASHSPNYRVISAALRNLACLAGLQDLSGSKSD

3DL_AA0884100.1 LGKAASEGTPPNLLVAEFSLKASIKTALASHSPNYRVISAALRNLACLAGLQDLSGSKSD

5BL_AA1305800.1 AVYGVYRQAYQIVVGLREGEYPCEEGQWLAVSAWNKSYLARRLNQASVGIKWMKMGLDLS

3B__AA0809860.1 AVYDVYRQAYQIVVGLREGEYPCEEGQWLAVSAWNKSYLARRLNQASVGIKWMKMGLDLS

3AL_AA0645950.1 AVYDVYRQAYQILVGLRDGEYPCEEGQWLAVTAWNKSYLARRLNQASVGIKWMKMGLDLS

3DL_AA0884100.1 AVYDVYRQAYQIMVGLRDGEYPCEEGQWLAVTAWNKSYLARRLNQASVGIKWMKMGLDLS

5BL_AA1305800.1 RHVESMKKYIADMEQCLEYFQKMIHSEAGEHALLGKNPDEGSRQEGAPSTSMSGSMSQPV

3B__AA0809860.1 RHVESMKKYTADMEQCLEYFQKMIHSEAGEHALLGKNPDECSQQEGAPSTRTRFIWCCTA

3AL_AA0645950.1 WHVESMKQYTADMEQCLEYFQKLFHSEAGEHALLGKNPDECSQQEGAPSTSMSGSMSQPV

3DL_AA0884100.1 RHVESMKQYTADMEQCLEYFQKLFHSEAGEHALLGKNPDECSQQEGAPSTREI-------

5BL_AA1305800.1 LV-

3B__AA0809860.1 IAS

3AL_AA0645950.1 LV-

3DL_AA0884100.1 ---

**Supplementary Fig. 2** Alignment of *TaZIP4-B2* splice variants in wild type Cadenza.

Splice_variant_1 ATGACGATCTCCGAGCTCTCTGAGCTCAAGCGGGTCGTCGATCTGTACGACGCCCCCGAC

Splice_variant_2 ATGACGATCTCCGAGCTCTCTGAGCTCAAGCGGGTCGTCGATCTGTACGACGCCCCCGAC

Splice_variant_3 ATGACGATCTCCGAGCTCTCTGAGCTCAAGCGGGTCGTCGATCTGTACGACGCCCCCGAC

Splice_variant_1 CCCTCCCCCCCGGAGAAGCTCGTCGCCGACTTCCGCCGCGTCCTCACCAACCTCGGCTCC

Splice_variant_2 CCCTCCCCCCCGGAGAAGCTCGTCGCCGACTTCCGCCGCGTCCTCACCAACCTCGGCTCC

Splice_variant_3 CCCTCCCCCCCGGAGAAGCTCGTCGCCGACTTCCGCCGCGTCCTCACCAACCTCGGCTCC

Splice_variant_1 GCCGCCTCCTCCCTCACCGACGCCTTACCCAGGCTACAGTTGTACAATCTCGGCAACCGC

Splice_variant_2 GCCGCCTCCTCCCTCACCGACGCCTTACCCAGGCTACAGTTGTACAATCTCGGCAACCGC

Splice_variant_3 GCCGCCTCCTCCCTCACCGACGCCTTACCCAGGCTACAGTTGTACAATCTCGGCAACCGC

Splice_variant_1 CTCTGGAAAGCCGTGGACAAGCGCGCCAACTCCGCCGCGCTGGCCCTGGGTCCCTCCGCC

Splice_variant_2 CTCTGGAAAGCCGTGGACAAGCGCGCCAACTCCGCCGCGCTGGCCCTGGGTCCCTCCGCC

Splice_variant_3 CTCTGGAAAGCCGTGGACAAGCGCGCCAACTCCGCCGCGCTGGCCCTGGGTCCCTCCGCC

Splice_variant_1 CGCGCCGCCGAGGCGGAGGTCAGGCAGGTGGCGCCGGAGCTGCTCCTCCTCGCCGGCTTC

Splice_variant_2 CGCGCCGCCGAGGCGGAGGTCAGGCAGGTGGCGCCGGAGCTGCTCCTCCTCGCCGGCTTC

Splice_variant_3 CGCGCCGCCGAGGCGGAGGTCAGGCAGGTGGCGCCGGAGCTGCTCCTCCTCGCCGGCTTC

Splice_variant_1 CCCGTCAAGGCCGCATCCTTCTTCCTCCGCGCCGGCCAGGGGTGGCTCGACCTCGGCCGC

Splice_variant_2 CCCGTCAAGGCCGCATCCTTCTTCCTCCGCGCCGGCCAGGGGTGGCTCGACCTCGGCCGC

Splice_variant_3 CCCGTCAAGGCCGCATCCTTCTTCCTCCGCGCCGGCCAGGGGTGGCTCGACCTCGGCCGC

Splice_variant_1 GTCGACCTCGCCACCGCCTGCTTCGACAAGGCCACGCCGCTCGTCTCCGCGCTCGCCACG

Splice_variant_2 GTCGACCTCGCCACCGCCTGCTTCGACAAGGCCACGCCGCTCGTCTCCGCGCTCGCCACG

Splice_variant_3 GTCGACCTCGCCACCGCCTGCTTCGACAAGGCCACGCCGCTCGTCTCCGCGCTCGCCACG

Splice_variant_1 GAGGATGAGCGACGCATCCTGCTCGGCCTCAACCTCGCGCGGGCGCGCGCGGCGTCCGAC

Splice_variant_2 GAGGATGAGCGACGCATCCTGCTCGGCCTCAACCTCGCGCGGGCGCGCGCGGCGTCCGAC

Splice_variant_3 GAGGATGAGCGACGCATCCTGCTCGGCCTCAACCTCGCGCGGGCGCGCGCGGCGTCCGAC

Splice_variant_1 GCGGGCGATCAGTCCCTCGCCGTCGCGCTGCTCGGCCGCTCCAAGTCCCTCGCGTCCCTC

Splice_variant_2 GCGGGCGATCAGTCCCTCGCCGTCGCGCTGCTCGGCCGCTCCAAGTCCCTCGCGTCCCTC

Splice_variant_3 GCGGGCGATCAGTCCCTCGCCGTCGCGCTGCTCGGCCGCTCCAAGTCCCTCGCGTCCCTC

Splice_variant_1 GCGTCCGCGCCCCGCAAGTATACCAGATCCCTCGCCGAGGGGTACCTGTACGTCGGCGAG

Splice_variant_2 GCGTCCGCGCCCCGCAAGTATACCAGATCCCTCGCCGAGGGGTACCTGTACGTCGGCGAG

Splice_variant_3 GCGTCCGCGCCCCGCAAGTATACCAGATCCCTCGCCGAGGGGTACCTGTACGTCGGCGAG

Splice_variant_1 GCCACGCTCTTCGCGAAGCCCTCCGACCCCGCCGTCGTAGCGTCCATGCTCCTCACCGAG

Splice_variant_2 GCCACGCTCTTCGCGAAGCCCTCCGACCCCGCCGTCGTAGCGTCCATGCTCCTCACCGAG

Splice_variant_3 GCCACGCTCTTCGCGAAGCCCTCCGACCCCGCCGTCGTAGCGTCCATGCTCCTCACCGAG

Splice_variant_1 GCGCTAGATCTCTTCCAGAAGCTGCCCCGCCCCCCAAAAAAAAAGAAGCTGGCCTCACCC

Splice_variant_2 GCGCTAGATCTCTTCCAGAAGCTGCCCCGCCCCCCAAAAAAAAAGAAGCTGGCCTCACCC

Splice_variant_3 GCGCTAGATCTCTTCCAGAAGCTGCCCCGCCCCCCAAAAAAAAAGAAGCTGGCCTCACCC

Splice_variant_1 TCCCCCTCCAGCTCGGAGCAAAAGGGTCGATGCCTCCGCTACCTTGCCCTCGAGCATCAC

Splice_variant_2 TCCCCCTCCAGCTCGGAGCAAAAGGGTCGATGCCTCCGCTACCTTGCCCTCGAGCATCAC

Splice_variant_3 TCCCCCTCCAGCTCGGAGCAAAAGGGTCGATGCCTCCGCTACCTTGCCCTCGAGCATCAC

Splice_variant_1 AAAGCCAAGGAACACGCTGACGTCCTGTTTTTTATCCATGCCTCGAGGGATTTAATTGGG

Splice_variant_2 AAAGCCAAGGAACACGCTGACGTCCTGTTTTTTATCCATGCCTCGAGGGATTTAATTGGG

Splice_variant_3 AAAGCCAAGGAACACGCTGACGTCCTGTTTTTTATCCATGCCTCGAGGGATTTAATTGGG

Splice_variant_1 GAGGCTGATGAGCACCCGAGCATGGGGTTCATGGCCCTGCACGCGTGGCTTGGTACCGGC

Splice_variant_2 GAGGCTGATGAGCACCCGAGCATGGGGTTCATGGCCCTGCACGCGTGGCTTGGTACCGGC

Splice_variant_3 GAGGCTGATGAGCACCCGAGCATGGGGTTCATGGCCCTGCACGCGTGGCTTGGTACCGGC

Splice_variant_1 AACTTGGCGGAGGCCGAGAGGGAGCTCGAGAGACTCATGGCCAACGCCAACGCATCAGAG

Splice_variant_2 AACTTGGCGGAGGCCGAGAGGGAGCTCGAGAGACTCATGGCCAACGCCAACGCATCAGAG

Splice_variant_3 AACTTGGCGGAGGCCGAGAGGGAGCTCGAGAGACTCATGGCCAACGCCAACGCATCAGAG

Splice_variant_1 AGTCTATGCGTGGGGGCTGCTGAGGTGTACCTTGCCTCCGCGGGGCCTGAGGCTGCACGC

Splice_variant_2 AGTCTATGCGTGGGGGCTGCTGAGGTGTACCTTGCCTCCGCGGGGCCTGAGGCTGCACGC

Splice_variant_3 AGTCTATGCGTGGGGGCTGCTGAGGTGTACCTTGCCTCCGCGGGGCCTGAGGCTGCACGC

Splice_variant_1 AAGGTGCTCGTTGCACTTGTTGCACGTTGCTGCGCGGGAGTTGCAGCTGCTGCTGTGAGA

Splice_variant_2 AAGGTGCTCGTTGCACTTGTTGCACGTTGCTGCGCGGGAGTTGCAGCTGCTGCTGTGAGA

Splice_variant_3 AAGGTGCTCGTTGCACTTGTTGCACGTTGCTGCGCGGGAGTTGCAGCTGCTGCTGTGAGA

Splice_variant_1 GTGGTGACCAAGGTAGTTGATTGTGGTATCGGTAGCCCTGGGCGCGCAAGGGCGATCGGT

Splice_variant_2 GTGGTGACCAAGGTAGTTGATTGTGGTATCGGTAGCCCTGGGCGCGCAAGGGCGATCGGT

Splice_variant_3 GTGGTGACCAAGGTAGTTGATTGTGGTATCGGTAGCCCTGGGCGCGCAAGGGCGATCGGT

Splice_variant_1 GAGCTCGTGTCGGATGAGAGGGTGGTCGCACTGTTCGATGGCCCGGCTAACACCAGTCAC

Splice_variant_2 GAGCTCGTGTCGGATGAGAGGGTGGTCGCACTGTTCGATGGCCCGGCTAACACCAGTCAC

Splice_variant_3 GAGCTCGTGTCGGATGAGAGGGTGGTCGCACTGTTCGATGGCCCGGCTAACACCAGTCAC

Splice_variant_1 CGTGGAGCAATGCATACACTGCTCTGGAA-------------------------------

Splice_variant_2 CGTGGAGCAATGCATACACTGCTCTGGAACTGGTCTGCACTCTCTTACACAAGTAATTTT

Splice_variant_3 CGTGGAGCAATGCATACACTGCTCTGGAA-------------------------------

Splice_variant_1 --------------------------------------------------------CTGC

Splice_variant_2 ACAGCAATTTTATGTACATATGATTTCTTGCTGAGGCTGACATTTGGAATTTGGGACAGC

Splice_variant_3 --------------------------------------------------------CTGC

Splice_variant_1 GGGTTTGAGCATTTCAATGCAAAGAACTACGATATATGTGCGGACTTGTTTGAGACATCG

Splice_variant_2 GGGTTTGAGCATTTCAATGCAAAGAACTACGATATATGTGCGGACTTGTTTGAGACATCG

Splice_variant_3 GGGTTTGAGCATTTCAATGCAAAGAACTACGATATATGTGCGGACTTGTTTGAGACATCG

Splice_variant_1 ATGCTTTATCTATCCCGTGAAGAGGGAAGCAGAGTCCGGCGAGCACAGTGCCTCCGAGTC

Splice_variant_2 ATGCTTTATCTATCCCGTGAAGAGGGAAGCAGAGTCCGGCGAGCACAGTGCCTCCGAGTC

Splice_variant_3 ATGCTTTATCTATCCCGTGAAGAGGGAAGCAGAGTCCGGCGAGCACAGTGCCTCCGAGTC

Splice_variant_1 CTTGCCGTTTGCTATCTAGCCCTTCAACGTCTGGATCGAGCACATGAGTTTGTCAATGAG

Splice_variant_2 CTTGCCGTTTGCTATCTAGCCCTTCAACGTCTGGATCGAGCACATGAGTTTGTCAATGAG

Splice_variant_3 CTTGCCGTTTGCTATCTAGCCCTTCAACGTCTGGATCGAGCACATGAGTTTGTCAATGAG

Splice_variant_1 GCTGACAAGGTGGAACACAATGCCCACTGTGCCTTTATGAAGATCAAAATTCATCTTCAG

Splice_variant_2 GCTGACAAGGTGGAACACAATGCCCACTGTGCCTTTATGAAGATCAAAATTCATCTTCAG

Splice_variant_3 GCTGACAAGGTGGAACACAATGCCCACTGTGCCTTTATGAAGATCAAAATTCATCTTCAG

Splice_variant_1 AAGAACGACGAGGACGAGGCTATCAAGCAGATAAAAACCATGATGGGTTGCATTGACTTC

Splice_variant_2 AAGAACGACGAGGACGAGGCTATCAAGCAGATAAAAACCATGATGGGTTGCATTGACTTC

Splice_variant_3 AAGAACGACGAGGACGAGGCTATCAAGCAGATAAAAACCATGATGGGTTGCATTGACTTC

Splice_variant_1 AATCCCGTGTTCCTAATGCTCACAACTCATGAGGCTATTGCCTGCAAGGCTGTCCGGGTA

Splice_variant_2 AATCCCGTGTTCCTAATGCTCACAACTCATGAGGCTATTGCCTGCAAGGCTGTCCGGGTA

Splice_variant_3 AATCCCGTGTTCCTAATGCTCACAACTCATGAGGCTATTGCCTGCAAGGCTGTCCGGGTA

Splice_variant_1 GCAGTTGCTTCATTGACCTTCCTTCTTGGTCTCTATTCTGCCGGAAAGCCTATGCCAGAG

Splice_variant_2 GCAGTTGCTTCATTGACCTTCCTTCTTGGTCTCTATTCTGCCGGAAAGCCTATGCCAGAG

Splice_variant_3 GCAGTTGCTTCATTGACCTTCCTTCTTGGTCTCTATTCTGCCGGAAAGCCTATGCCAGAG

Splice_variant_1 CGTGAGGTTACTGTCCTCCGCACTCTTATTGAGCTCCTCCGTCGTGAGCAAGGTACTGAG

Splice_variant_2 CGTGAGGTTACTGTCCTCCGCACTCTTATTGAGCTCCTCCGTCGTGAGCAAGGTACTGAG

Splice_variant_3 CGTGAGGTTACTGTCCTCCGCACTCTTATTGAGCTCCTCCGTCGTGAGCAAGGTACTGAG

Splice_variant_1 GATGAGATCCTTAAGTACTCGAGACGTGCCAAGCTACGGATGTCTAACCTTGGCGTGGAA

Splice_variant_2 GATGAGATCCTTAAGTACTCGAGACGTGCCAAGCTACGGATGTCTAACCTTGGCGTGGAA

Splice_variant_3 GATGAGATCCTTAAGTACTCGAGACGTGCCAAGCTACGGATGTCTAACCTTGGCGTGGAA

Splice_variant_1 GGCTTTTTTGGCAATGGACCTGTTGGGGCGCGTGAACTGAACTGGTTTGCAGGCAATAGC

Splice_variant_2 GGCTTTTTTGGCAATGGACCTGTTGGGGCGCGTGAACTGAACTGGTTTGCAGGCAATAGC

Splice_variant_3 GGCTTTTTTGGCAATGGACCTGTTGGGGCGCGTGAACTGAACTGGTTTGCAGGCAATAGC

Splice_variant_1 TGGAATATGGGTAGAAGGGTGGCTAAGGAGCAAAAGTATGATCTTAGTGCTGAGTTCTTT

Splice_variant_2 TGGAATATGGGTAGAAGGGTGGCTAAGGAGCAAAAGTATGATCTTAGTGCTGAGTTCTTT

Splice_variant_3 TGGAATATGGGTAGAAGGGTGGCTAAGGAGCAAAAGTATGATCTTAGTGCTGAGTTCTTT

Splice_variant_1 GAGCTTGCAGCAGAGTTCTTTGGTGGTGCAAGTAATGATGAGGGTGATGGAAATCACCCC

Splice_variant_2 GAGCTTGCAGCAGAGTTCTTTGGTGGTGCAAGTAATGATGAGGGTGATGGAAATCACCCC

Splice_variant_3 GAGCTTGCAGCAGAGTTCTTTGGTGGTGCAAGTAATGATGAGGGTGATGGAAATCACCCC

Splice_variant_1 ACACTTTGCAAAGCGTTAATCATGAGTGTCACTTCCATGCTTAAAGCTGAGGAGCTAAAC

Splice_variant_2 ACACTTTGCAAAGCGTTAATCATGAGTGTCACTTCCATGCTTAAAGCTGAGGAGCTAAAC

Splice_variant_3 ACACTTTGCAAAGCGTTAATCATGAGTGTCACTTCCATGCTTAAAGCTGAGGAGCTAAAC

Splice_variant_1 AATTCTCCCTTGTTGGATTCTGATGTTAAAAAAGGTGTTGAGATGCTCAGCAGAGCTGGC

Splice_variant_2 AATTCTCCCTTGTTGGATTCTGATGTTAAAAAAGGTGTTGAGATGCTCAGCAGAGCTGGC

Splice_variant_3 AATTCTCCCTTGTTGGATTCTGATGTTAAAAAAGGTGTTGAGATGCTCAGCAGAGCTGGC

Splice_variant_1 AAGCTATTACCTTCAATCTGGCCCTCGGGTTCAGTCGCCTCTGATCAGGCTGAGGCCAAC

Splice_variant_2 AAGCTATTACCTTCAATCTGGCCCTCGGGTTCAGTCGCCTCTGATCAGGCTGAGGCCAAC

Splice_variant_3 AAGGTAAACATG--------------GGGTTTAACAAATACTAA----------------

Splice_variant_1 ATCTTTCTGTTTCTTCATACCTTCTACTCCTACCAACTCCTTGACAGGATGGACACCAGC

Splice_variant_2 ATCTTTCTGTTTCTTCATACCTTCTACTCCTACCAACTCCTTGACAGGATGGACACCAGC

Splice_variant_3 ------------------------------------------------------------

Splice_variant_1 GCACATCCTCAGCAGCTCCAACTAGTCAAGAACTTCGCTTCCTCTAAAGCATGCACGCCG

Splice_variant_2 GCACATCCTCAGCAGCTCCAACTAGTCAAGAACTTCGCTTCCTCTAAAGCATGCACGCCG

Splice_variant_3 ------------------------------------------------------------

Splice_variant_1 TCCCATCTTCTCGTACTTGGGAAAGCTGCTTCTGAAGGCACCCCACCAAACCTGCTGGTT

Splice_variant_2 TCCCATCTTCTCGTACTTGGGAAAGCTGCTTCTGAAGGCACCCCACCAAACCTGCTGGTT

Splice_variant_3 ------------------------------------------------------------

Splice_variant_1 GCTGAATTTTCACTGAAGGCTAGCATCAAGACCGCCCTTGCTTCTCACTCCCCAAACTAC

Splice_variant_2 GCTGAATTTTCACTGAAGGCTAGCATCAAGACCGCCCTTGCTTCTCACTCCCCAAACTAC

Splice_variant_3 ------------------------------------------------------------

Splice_variant_1 AGGGTAATCAGTGCTGCCCTCAGGAACCTAGCCTGCCTTGCTGGCTTGCAAGACTTAAGT

Splice_variant_2 AGGGTAATCAGTGCTGCCCTCAGGAACCTAGCCTGCCTTGCTGGCTTGCAAGACTTAAGT

Splice_variant_3 ------------------------------------------------------------

Splice_variant_1 GGTAGCAAGAGTGATGCAGTGTATGGTGTATATCGACAAGCTTACCAGATCGTGGTTGGA

Splice_variant_2 GGTAGCAAGAGTGATGCAGTGTATGGTGTATATCGACAAGCTTACCAGATCGTGGTTGGA

Splice_variant_3 ------------------------------------------------------------

Splice_variant_1 CTGAGAGAGGGCGAATATCCATGTGAGGAAGGGCAGTGGCTTGCAGTGTCTGCCTGGAAC

Splice_variant_2 CTGAGAGAGGGCGAATATCCATGTGAGGAAGGGCAGTGGCTTGCAGTGTCTGCCTGGAAC

Splice_variant_3 ------------------------------------------------------------

Splice_variant_1 AAGTCATATTTGGCTAGGCGGCTTAATCAAGCTTCAGTTGGTATAAAATGGATGAAGATG

Splice_variant_2 AAGTCATATTTGGCTAGGCGGCTTAATCAAGCTTCAGTTGGTATAAAATGGATGAAGATG

Splice_variant_3 ------------------------------------------------------------

Splice_variant_1 GGATTAGATCTCTCTCGGCATGTTGAGAGCATGAAGAAGTATATAGCAGACATGGAGCAA

Splice_variant_2 GGATTAGATCTCTCTCGGCATGTTGAGAGCATGAAGAAGTATATAGCAGACATGGAGCAA

Splice_variant_3 ------------------------------------------------------------

Splice_variant_1 TGCCTTGAGTACTTCCAGAAAATGATTCATAGTGAAGCTGGTGAACATGCTTTGCTCGGA

Splice_variant_2 TGCCTTGAGTACTTCCAGAAAATGATTCATAGTGAAGCTGGTGAACATGCTTTGCTCGGA

Splice_variant_3 ------------------------------------------------------------

Splice_variant_1 AAAAACCCTGATGAAGGTAGTCGGCAAGAGGGGGCACCAAGCACAAGTATGTCTGGTAGC

Splice_variant_2 AAAAACCCTGATGAAGGTAGTCGGCAAGAGGGGGCACCAAGCACAAGTATGTCTGGTAGC

Splice_variant_3 ------------------------------------------------------------

Splice_variant_1 ATGTCTCAGCCTGTCTTAGTATAG

Splice_variant_2 ATGTCTCAGCCTGTCTTAGTATAG

Splice_variant_3 ------------------------

**Supplementary Fig. 3** Alignment of *TaZIP4-B2* coding DNA sequences from wild type Cadenza (WTC) and mutant Cadenza lines (MCL). Both missense and nonsense mutants are highlighted. Primer sequences used to follow mutated genes during crossing are underlined. All primers are shown in direction 5´ 🡪 3´.

WTC CAGTTAAAACGAGCGGCAGTTTAGCACTGTTGTGGTTGCCGCAGCAGGGGAAGCCGGCCA 60

MCL CAGTTAAAACGAGCGGCAGTTTAGCACTGTTGTGGTTGCCGCAGCAGGGGAAGCCGGCCA 60

************************************************************

WTC TGGCTACGCGCGAAGGCAGACAACGGCTCACGGCGGCGGAGTGAGCCAGAGGCGCGCCAG 120

MCL TGGCTACGCGCGAAGGCAGACAACGGCTCACGGCGGCGGAGTGAGCCAGAGGCGCGCCAG 120

************************************************************

WTC CCACAGTGGCTTGATGCAACGGGGAAGACCGTGGTGATGGTGAGGCGACGATGGCCGGTG 180

MCL CCACAGTGGCTTGATGCAACGGGGAAGACCGTGGTGATGGTGAGGCGACGATGGCCGGTG 180

************************************************************

WTC AGGCCACGCGTGCCTAAGCAGGCGTCGGGCTGCTCGCGCCGGGGTGGCTCAGTGGCTCGG 240

MCL AGGCCACGCGTGCCTAAGCAGGCGTCGGGCTGCTCGCGCCGGGGTGGCTCAGTGGCTCGG 240

************************************************************

WTC CGACCGGCAGTTCGAACGACGAGGGCGCGACGGCGGCGACCGGCCCAGAGGTGATGGGAG 300

MCL CGACCGGCAGTTCGAACGACGAGGGCGCGACGGCGGCGACCGGCCCAGAGGTGATGGGAG 300

************************************************************

WTC GTTGTGGCGGGTTGGCGAAAGCGATGGCACGGCCGGCTTCGACGAGGTTGGAACAAGGGA 360

MCL GTTGTGGCGGGTTGGCGAAAGCGATGGCACGGCCGGCTTCGACGAGGTTGGAACAAGGGA 360

************************************************************

WTC GGCGCCTGGTTTAGCGGCGGCGACTCAGGGCCAGCAGAGCGGCGCATCCGACGGAGATGG 420

MCL GGCGCCTGGTTTAGCGGCGGCGACTCAGGGCCAGCAGAGCGGCGCATCCGACGGAGATGG 420

************************************************************

WTC CTAGCGGAGCGAGCCGCGGCGACGGGTTGACTCAGGTCACAAGAAGTCAACTCCCATCGC 480

MCL CTAGCGGAGCGAGCCGCGGCGACGGGTTGACTCAGGTCACAAGAAGTCAACTCCCATCGC 480

************************************************************

WTC GGCTCAGCAAACAGGCGTGGAGGCGCTGCGGCGACTCCGGCGGAGGATCGAATCCACGTC 540

MCL GGCTCAGCAAACAGGCGTGGAGGCGCTGCGGCGACTCCGGCGGAGGATCGAATCCACGTC 540

************************************************************

WTC CGCTTTCCGTGTCGTGGCCGGCTTAATCGACTCTCGTCCGTGTCCTCCTTCCAGTCGTGG 600

MCL CGCTTTCCGTGTCGTGGCCGGCTTAATCGACTCTCGTCCGTGTCCTCCTTCCAGTCGTGG 600

************************************************************

WTC TTGGTTTGACTCTTTTCTTTTCTTTTGACTTGATTGTTTCTCAGATCGGACTCGTACTAG 660

MCL TTGGTTTGACTCTTTTCTTTTCTTTTGACTTGATTGTTTCTCAGATCGGACTCGTACTAG 660

************************************************************

WTC CAATACTTAATAGCTTTGTTTGTACGCCCATAGGAAATGGGGATCAAACTCTGTAGCTTG 720

MCL CAATACTTAATAGCTTTGTTTGTACGCCCATAGGAAATGGGGATCAAACTCTGTAGCTTG 720

************************************************************

WTC ACATGATGAGTTACCAGTACTTGATAATTTGGTATGGGCTATCTGACTAGCTCTGTCTCC 780

MCL ACATGATGAGTTACCAGTACTTGATAATTTGGTATGGGCTATCTGACTAGCTCTGTCTCC 780

************************************************************

WTC ATGCGGACGGGTTGATGATTCTGATGGATTTGATGTGTGCGCTTTCAGCGGCGTTGCTTC 840

MCL ATGCGGACGGGTTGATGATTCTGATGGATTTGATGTGTGCGCTTTCAGCGGCGTTGCTTC 840

************************************************************

WTC TGATTGTCCATCCGTCCTGGCGCGAAGACGGCCATAGCAGAGGAGAGCAGCCGCGGGTGA 900

MCL TGATTGTCCATCCGTCCTGGCGCGAAGACGGCCATAGCAGAGGAGAGCAGCCGCGGGTGA 900

************************************************************

WTC CGATGATTGCAGCAAAAGGCTGTCTTCGTCGTTGCAGTCGTAGCTCCGACTTGAATGGAT 960

MCL CGATGATTGCAGCAAAAGGCTGTCTTCGTCGTTGCAGTCGTAGCTCCGACTTGAATGGAT 960

************************************************************

WTC TTAGCAAAAAAAAACGCTGGTTCCAGTACGCCGGGACGCTGGTTTCAGCATGCGGCCGTC 1020

MCL TTAGCAAAAAAAAACGCTGGTTCCAGTACGCCGGGACGCTGGTTTCAGCATGCGGCCGTC 1020

************************************************************

WTC ACGGTCGCAGCTCGCCGGACGACGCATTGGAGGATGAGAGGGAAAAAAAGCAGCCGTGAT 1080

MCL ACGGTCGCAGCTCGCCGGACGACGCATTGGAGGATGAGAGGGAAAAAAAGCAGCCGTGAT 1080

************************************************************

WTC GGGCCAAGGGGCATGTGGTGGGCCGAGGAGGAAATCCAACAACGCAAACAATTAATGAAT 1140

MCL GGGCCAAGGGGCATGTGGTGGGCCGAGGAGGAAATCCAACAACGCAAACAATTAATGAAT 1140

************************************************************

WTC CTGTAGACCTGTTTTTTTTTCTTTTTTTTTTGAAGTTGAATCTGTAGACCTGTTAGCCAT 1200

MCL CTGTAGACCTGTTTTTTTTTCTTTTTTTTTTGAAGTTGAATCTGTAGACCTGTTAGCCAT 1200

************************************************************

WTC AGAATCGCTGGGCGATCCAGCGGTACGCGAGCGACCGGCCAAAGCTTCCCAAAAAAAAAC 1260

MCL AGAATCGCTGGGCGATCCAGCGGTACGCGAGCGACCGGCCAAAGCTTCCCAAAAAAAAAC 1260

************************************************************

WTC GAAGGAAGGAAGGAAGGAAGGACCGGCACGAACCCTCGCCCCATCCCCCGTTCCCCACGC 1320

MCL GAAGGAAGGAAGGAAGGAAGGACCGGCACGAACCCTCGCCCCATCCCCCGTTCCCCACGC 1320

************************************************************

Forward primer (Cadenza1691)

WTC CTCGCCTCGCCGCGCCGCATCCCGCCGGAACCCTAGCCCCCGCCGCCATGACGATCTCCG 1380

MCL CTCGCCTCGCCGCGCCGCATCCCGCCGGAACCCTAGCCCCCGCCGCCATGACGATCTCCG 1380

************************************************************

WTC AGCTCTCTGAGCTCAAGCGGGTCGTCGATCTGTACGACGCCCCCGACCCCTCCCCCCCGG 1440

MCL AGCTCTCTGAGCTCAAGCGGGTCGTCGATCTGTACGACGCCCCCGACCCCTCCCCCCCGG 1440

************************************************************

WTC AGAAGCTCGTCGCCGACTTCCGCCGCGTCCTCACCAACCTCGGCTCCGCCGCCTCCTCCC 1500

MCL AGAAGCTCGTCGCCGACTTCCGCCGCGTCCTCACCAACCTCGGCTCCGCCGCCTCCTCCC 1500

************************************************************

WTC TCACCGACGCCTTACCCAGGCTACAGTTGTACAATCTCGGCAACCGCCTCTGGAAAGCCG 1560

MCL TCACCGACGCCTTACCCAGGCTACAGTTGTACAATCTCGGCAACCGCCTCTGGAAAGCCG 1560

************************************************************

WTC TGGACAAGCGCGCCAACTCCGCCGCGCTGGCCCTGGGTCCCTCCGCCCGCGCCGCCGAGG 1620

MCL TGGACAAGCGCGCCAACTCCGCCGCGCTGGCCCTGGGTCCCTCCGCCCGCGCCGCCGAGG 1620

************************************************************

WTC CGGAGGTCAGGCAGGTGGCGCCGGAGCTGCTCCTCCTCGCCGGCTTCCCCGTCAAGGCCG 1680

MCL CGGAGGTCAGGCAGGTGGCGCCGGAGCTGCTCCTCCTCGCCGGCTTCCCCGTCAAGGCCG 1680

************************************************************

WTC CATCCTTCTTCCTCCGCGCCGGCCAGGGGTGGCTCGACCTCGGCCGCGTCGACCTCGCCA 1740

MCL CATCCTTCTTCCTCCGCGCCGGCCAGGGGTGGCTCGACCTCGGCCGCGTCGACCTCGCCA 1740

************************************************************

WTC CCGCCTGCTTCGACAAGGCCACGCCGCTCGTCTCCGCGCTCGCCACGGAGGATGAGCGAC 1800

MCL CCGCCTGCTTCGACAAGGCCACGCCGCTCGTCTCCGCGCTCGCCACGGAGGATGAGCGAC 1800

************************************************************

WTC GCATCCTGCTCGGCCTCAACCTCGCGCGGGCGCGCGCGGCGTCCGACGCGGGCGATCAGT 1860

MCL GCATCCTGCTCGGCCTCAACCTCGCGCGGGCGCGCGCGGCGTCCGACGCGGGCGATCAGT 1860

************************************************************

Missense mutation (position 1867) Reverse primer (Cadenza1691)

WTC CCCTCG**C**CGTCGCGCTGCTCGGCCGCTCCAAGTCCCTCGCGTCCCTCGCGTCCGCGCCCC 1920

MCL CCCTCG**T**CGTCGCGCTGCTCGGCCGCTCCAAGTCCCTCGCGTCCCTCGCGTCCGCGCCCC 1920

****** *****************************************************

WTC GCAAGTATACCAGATCCCTCGCCGAGGGGTACCTGTACGTCGGCGAGGCCACGCTCTTCG 1980

MCL GCAAGTATACCAGATCCCTCGCCGAGGGGTACCTGTACGTCGGCGAGGCCACGCTCTTCG 1980

************************************************************

WTC CGAAGCCCTCCGACCCCGCCGTCGTAGCGTCCATGCTCCTCACCGAGGCGCTAGATCTCT 2040

MCL CGAAGCCCTCCGACCCCGCCGTCGTAGCGTCCATGCTCCTCACCGAGGCGCTAGATCTCT 2040

************************************************************

WTC TCCAGAAGCTGCCCCGCCCCCCAAAAAAAAAGAAGCTGGCCTCACCCTCCCCCTCCAGCT 2100

MCL TCCAGAAGCTGCCCCGCCCCCCAAAAAAAAAGAAGCTGGCCTCACCCTCCCCCTCCAGCT 2100

************************************************************

WTC CGGAGCAAAAGGGTCGATGCCTCCGCTACCTTGCCCTCGAGCATCACAAAGCCAAGGAAC 2160

MCL CGGAGCAAAAGGGTCGATGCCTCCGCTACCTTGCCCTCGAGCATCACAAAGCCAAGGAAC 2160

************************************************************

WTC ACGCTGACGTCCTGTTTTTTATCCATGCCTCGAGGGATTTAATTGGGGAGGCTGATGAGC 2220

MCL ACGCTGACGTCCTGTTTTTTATCCATGCCTCGAGGGATTTAATTGGGGAGGCTGATGAGC 2220

************************************************************

WTC ACCCGAGCATGGGGTTCATGGCCCTGCACGCGTGGCTTGGTACCGGCAACTTGGCGGAGG 2280

MCL ACCCGAGCATGGGGTTCATGGCCCTGCACGCGTGGCTTGGTACCGGCAACTTGGCGGAGG 2280

************************************************************

WTC CCGAGAGGGAGCTCGAGAGACTCATGGCCAACGCCAACGCATCAGAGAGTCTATGCGTGG 2340

MCL CCGAGAGGGAGCTCGAGAGACTCATGGCCAACGCCAACGCATCAGAGAGTCTATGCGTGG 2340

************************************************************

WTC GGGCTGCTGAGGTGTACCTTGCCTCCGCGGGGCCTGAGGCTGCACGCAAGGTGCTCGTTG 2400

MCL GGGCTGCTGAGGTGTACCTTGCCTCCGCGGGGCCTGAGGCTGCACGCAAGGTGCTCGTTG 2400

************************************************************

WTC CACTTGTTGCACGTTGCTGCGCGGGAGTTGCAGCTGCTGCTGTGAGAGTGGTGACCAAGG 2460

MCL CACTTGTTGCACGTTGCTGCGCGGGAGTTGCAGCTGCTGCTGTGAGAGTGGTGACCAAGG 2460

************************************************************

WTC TAGTTGATTGTGGTATCGGTAGCCCTGGGCGCGCAAGGGCGATCGGTGAGCTCGTGTCGG 2520

MCL TAGTTGATTGTGGTATCGGTAGCCCTGGGCGCGCAAGGGCGATCGGTGAGCTCGTGTCGG 2520

************************************************************

WTC ATGAGAGGGTGGTCGCACTGTTCGATGGCCCGGCTAACACCAGTCACCGTGGAGCAATGC 2580

MCL ATGAGAGGGTGGTCGCACTGTTCGATGGCCCGGCTAACACCAGTCACCGTGGAGCAATGC 2580

************************************************************

WTC ATACACTGCTCTGGAACTGGTCTGCACTCTCTTACACAAGTAATTTTACAGCAATTTTAT 2640

MCL ATACACTGCTCTGGAACTGGTCTGCACTCTCTTACACAAGTAATTTTACAGCAATTTTAT 2640

************************************************************

WTC GTACATATGATTTCTTGCTGAGGCTGACATTTGGAATTTGGGACAGCGGGTTTGAGCATT 2700

MCL GTACATATGATTTCTTGCTGAGGCTGACATTTGGAATTTGGGACAGCGGGTTTGAGCATT 2700

************************************************************

WTC TCAATGCAAAGAACTACGATATATGTGCGGACTTGTTTGAGACATCGATGCTTTATCTAT 2760

MCL TCAATGCAAAGAACTACGATATATGTGCGGACTTGTTTGAGACATCGATGCTTTATCTAT 2760

************************************************************

WTC CCCGTGAAGAGGGAAGCAGAGTCCGGCGAGCACAGTGCCTCCGAGTCCTTGCCGTTTGCT 2820

MCL CCCGTGAAGAGGGAAGCAGAGTCCGGCGAGCACAGTGCCTCCGAGTCCTTGCCGTTTGCT 2820

************************************************************

WTC ATCTAGCCCTTCAACGTCTGGATCGAGCACATGAGTTTGTCAATGAGGCTGACAAGGTAT 2880

MCL ATCTAGCCCTTCAACGTCTGGATCGAGCACATGAGTTTGTCAATGAGGCTGACAAGGTAT 2880

************************************************************

WTC TGATCCCTCTCCTCATCACTTCATTAAATAATCTTGCCTATTGCTTCTTACGATTTGCTC 2940

MCL TGATCCCTCTCCTCATCACTTCATTAAATAATCTTGCCTATTGCTTCTTACGATTTGCTC 2940

************************************************************

WTC AAAATGTGTATCTGTAGGTGGAACACAATGCCCACTGTGCCTTTATGAAGGTAATAAATA 3000

MCL AAAATGTGTATCTGTAGGTGGAACACAATGCCCACTGTGCCTTTATGAAGGTAATAAATA 3000

************************************************************

WTC TACATGCTAATTGTTCAGGGGTCTCATTTAACTCTGGTATTGTTTGCTAACCCAGAAGAA 3060

MCL TACATGCTAATTGTTCAGGGGTCTCATTTAACTCTGGTATTGTTTGCTAACCCAGAAGAA 3060

************************************************************

WTC TTGTGCAGATCAAAATTCATCTTCAGAAGAACGACGAGGACGAGGCTATCAAGCAGATAA 3120

MCL TTGTGCAGATCAAAATTCATCTTCAGAAGAACGACGAGGACGAGGCTATCAAGCAGATAA 3120

************************************************************

Forward primer (Cadenza0348)

WTC AAACCATGATGGGTTGCATTGACTTCAATCCCGTGTTCCTAATGCTCACAACTCATGAGG 3180

MCL AAACCATGATGGGTTGCATTGACTTCAATCCCGTGTTCCTAATGCTCACAACTCATGAGG 3180

************************************************************

WTC CTATTGCCTGCAAGGCTGTCCGGGTAGCAGTTGCTTCATTGACCTTCCTTCTTGGTCTCT 3240

MCL CTATTGCCTGCAAGGCTGTCCGGGTAGCAGTTGCTTCATTGACCTTCCTTCTTGGTCTCT 3240

************************************************************

WTC ATTCTGCCGGAAAGCCTATGCCAGAGCGTGAGGTTACTGTCCTCCGCACTCTTATTGAGC 3300

MCL ATTCTGCCGGAAAGCCTATGCCAGAGCGTGAGGTTACTGTCCTCCGCACTCTTATTGAGC 3300

************************************************************

WTC TCCTCCGTCGTGAGCAAGGTACTGAGGATGAGATCCTTAAGTACTCGAGACGTGCCAAGC 3360

MCL TCCTCCGTCGTGAGCAAGGTACTGAGGATGAGATCCTTAAGTACTCGAGACGTGCCAAGC 3360

************************************************************

WTC TACGGATGTCTAACCTTGGCGTGGAAGGCTTTTTTGGCAATGGACCTGTTGGGGCGCGTG 3420

MCL TACGGATGTCTAACCTTGGCGTGGAAGGCTTTTTTGGCAATGGACCTGTTGGGGCGCGTG 3420

************************************************************

Nonsense mutation (position 3449)

WTC AACTGAACTGGTTTGCAGGCAATAGCTG**G**AATATGGGTAGAAGGGTGGCTAAGGAGCAAA 3480

MCL AACTGAACTGGTTTGCAGGCAATAGCTG**A**AATATGGGTAGAAGGGTGGCTAAGGAGCAAA 3480

**************************** *******************************

WTC AGTATGATCTTAGTGCTGAGTTCTTTGAGCTTGCAGCAGAGTTCTTTGGTGGTGCAAGTA 3540

MCL AGTATGATCTTAGTGCTGAGTTCTTTGAGCTTGCAGCAGAGTTCTTTGGTGGTGCAAGTA 3540

************************************************************

WTC ATGATGAGGGTGATGGAAATCACCCCACACTTTGCAAAGCGTTAATCATGAGTGTCACTT 3600

MCL ATGATGAGGGTGATGGAAATCACCCCACACTTTGCAAAGCGTTAATCATGAGTGTCACTT 3600

************************************************************

WTC CCATGCTTAAAGCTGAGGAGCTAAACAATTCTCCCTTGTTGGATTCTGATGTTAAAAAAG 3660

MCL CCATGCTTAAAGCTGAGGAGCTAAACAATTCTCCCTTGTTGGATTCTGATGTTAAAAAAG 3660

************************************************************

WTC GTGTTGAGATGCTCAGCAGAGCTGGCAAGGTAAACATGGGGTTTAACAAATACTAAAAGT 3720

MCL GTGTTGAGATGCTCAGCAGAGCTGGCAAGGTAAACATGGGGTTTAACAAATACTAAAAGT 3720

************************************************************

WTC TACTACCCCTTTGTGTATGTTCTAAAGTGGTTCAGTTTTCTCTACTATTATTCTGATTCA 3780

MCL TACTACCCCTTTGTGTATGTTCTAAAGTGGTTCAGTTTTCTCTACTATTATTCTGATTCA 3780

************************************************************

WTC GTTCTATTGATGTGCAGCTATTACCTTCAATCTGGCCCTCGGGTTCAGTCGCCTCTGATC 3840

MCL GTTCTATTGATGTGCAGCTATTACCTTCAATCTGGCCCTCGGGTTCAGTCGCCTCTGATC 3840

************************************************************

WTC AGGCTGAGGCCAACATCTTTCTGTTTCTTCATACCTTCTACTCCTACCAACTCCTTGACA 3900

MCL AGGCTGAGGCCAACATCTTTCTGTTTCTTCATACCTTCTACTCCTACCAACTCCTTGACA 3900

************************************************************

WTC GGATGGACACCAGCGCACATCCTCAGCAGCTCCAACTAGTCAAGAACTTCGCTTCCTCTA 3960

MCL GGATGGACACCAGCGCACATCCTCAGCAGCTCCAACTAGTCAAGAACTTCGCTTCCTCTA 3960

************************************************************

WTC AAGCATGCACGCCGTCCCATCTTCTCGTACTTGGGAAAGCTGCTTCTGAAGGCACCCCAC 4020

MCL AAGCATGCACGCCGTCCCATCTTCTCGTACTTGGGAAAGCTGCTTCTGAAGGCACCCCAC 4020

************************************************************

WTC CAAACCTGCTGGTTGCTGAATTTTCACTGAAGGCTAGCATCAAGACCGCCCTTGCTTCTC 4080

MCL CAAACCTGCTGGTTGCTGAATTTTCACTGAAGGCTAGCATCAAGACCGCCCTTGCTTCTC 4080

************************************************************

WTC ACTCCCCAAACTACAGGGTAATCAGTGCTGCCCTCAGGAACCTAGCCTGCCTTGCTGGCT 4140

MCL ACTCCCCAAACTACAGGGTAATCAGTGCTGCCCTCAGGAACCTAGCCTGCCTTGCTGGCT 4140

************************************************************

WTC TGCAAGACTTAAGTGGTAGCAAGAGTGATGCAGTGTATGGTGTATATCGACAAGCTTACC 4200

MCL TGCAAGACTTAAGTGGTAGCAAGAGTGATGCAGTGTATGGTGTATATCGACAAGCTTACC 4200

************************************************************

WTC AGATCGTGGTTGGACTGAGAGAGGGCGAATATCCATGTGAGGAAGGGCAGTGGCTTGCAG 4260

MCL AGATCGTGGTTGGACTGAGAGAGGGCGAATATCCATGTGAGGAAGGGCAGTGGCTTGCAG 4260

************************************************************

WTC TGTCTGCCTGGAACAAGTCATATTTGGCTAGGCGGCTTAATCAAGCTTCAGTTGGTATAA 4320

MCL TGTCTGCCTGGAACAAGTCATATTTGGCTAGGCGGCTTAATCAAGCTTCAGTTGGTATAA 4320

************************************************************

WTC AATGGATGAAGATGGGATTAGATCTCTCTCGGCATGTTGAGAGCATGAAGAAGTATATAG 4380

MCL AATGGATGAAGATGGGATTAGATCTCTCTCGGCATGTTGAGAGCATGAAGAAGTATATAG 4380

************************************************************

WTC CAGACATGGAGCAATGCCTTGAGTACTTCCAGAAAATGATTCATAGTGAAGCTGGTGAAC 4440

MCL CAGACATGGAGCAATGCCTTGAGTACTTCCAGAAAATGATTCATAGTGAAGCTGGTGAAC 4440

************************************************************

Reverse primer (Cadenza0348)

WTC ATGCTTTGCTCGGAAAAAACCCTGATGAAGGTAGTCGGCAAGAGGGGGCACCAAGCACAA 4500

MCL ATGCTTTGCTCGGAAAAAACCCTGATGAAGGTAGTCGGCAAGAGGGGGCACCAAGCACAA 4500

************************************************************

WTC GTATGTCTGGTAGCATGTCTCAGCCTGTCTTAGTATAGTATAGATGCTTCTAAACGGGGC 4560

MCL GTATGTCTGGTAGCATGTCTCAGCCTGTCTTAGTATAGTATAGATGCTTCTAAACGGGGC 4560

************************************************************

WTC AGTTCAAAACCAAGAGATAAATAGATCATTGAGTTATGTACTTGAGTAATTGCGTATGAC 4620

MCL AGTTCAAAACCAAGAGATAAATAGATCATTGAGTTATGTACTTGAGTAATTGCGTATGAC 4620

************************************************************

WTC CCGTCAAATATTGTCTGATTTACATCTCTAGCAAGCATGTGGATTCTTTGTCGATAGTAA 4680

MCL CCGTCAAATATTGTCTGATTTACATCTCTAGCAAGCATGTGGATTCTTTGTCGATAGTAA 4680

************************************************************

WTC CCATGTCTGTGAATTTAATGCATGCTGATGTGTAGGAGAGATGACTGATTGCTTGTAATA 4740

MCL CCATGTCTGTGAATTTAATGCATGCTGATGTGTAGGAGAGATGACTGATTGCTTGTAATA 4740

************************************************************

WTC CAGTACTGTAACATTGATTTGGGAACCTACTGGATTTGTTCAGGCAACCTTTATGTCCAT 4800

MCL CAGTACTGTAACATTGATTTGGGAACCTACTGGATTTGTTCAGGCAACCTTTATGTCCAT 4800

************************************************************

WTC TTTCTGTCAGATTTGACTAATCTCCTGATGTCCCTATCATCCAAACTATTATAGTGGAAA 4860

MCL TTTCTGTCAGATTTGACTAATCTCCTGATGTCCCTATCATCCAAACTATTATAGTGGAAA 4860

************************************************************

WTC ACTGGAGAGGGGCAGAAATACATAAAGAAGAGTAAGGACTCACTGAAACCTTCTATAGTC 4920

MCL ACTGGAGAGGGGCAGAAATACATAAAGAAGAGTAAGGACTCACTGAAACCTTCTATAGTC 4920

************************************************************

WTC TACACAACCATGTTAGGCCAACTCCACCGCGCGACCCCAAACGGACGTCCGTTTTGTCCG 4980

MCL TACACAACCATGTTAGGCCAACTCCACCGCGCGACCCCAAACGGACGTCCGTTTTGTCCG 4980

************************************************************

WTC GATTCTATCCGTTTGGGTAGGGGTTTGGGGTCGTATCCGGGCCTGTCCTGGTATGCGGTG 5040

MCL GATTCTATCCGTTTGGGTAGGGGTTTGGGGTCGTATCCGGGCCTGTCCTGGTATGCGGTG 5040

************************************************************

WTC GCCGTGCGCCCAGCGCGCGGCCGCATCCATTTGCCCCATCCTGTCCGTGAGGGCCAAAAA 5100

MCL GCCGTGCGCCCAGCGCGCGGCCGCATCCATTTGCCCCATCCTGTCCGTGAGGGCCAAAAA 5100

************************************************************

WTC TGCCTAAATTTGCATCAAACTACTTCCAACCCAAATATTTGTCTGAAAATTAAAATAGTT 5160

MCL TGCCTAAATTTGCATCAAACTACTTCCAACCCAAATATTTGTCTGAAAATTAAAATAGTT 5160

************************************************************

WTC TTACAACCCAATTGAAATTGTCTTAAATAAAATAGTTTTACAACCAAATCGAAATTGTCT 5220

MCL TTACAACCCAATTGAAATTGTCTTAAATAAAATAGTTTTACAACCAAATCGAAATTGTCT 5220

************************************************************

WTC TGACTGAACATAAATTGGACCAATACATCTATTGGTTGCCAATGTGATCCCAGACGTGCT 5280

MCL TGACTGAACATAAATTGGACCAATACATCTATTGGTTGCCAATGTGATCCCAGACGTGCT 5280

************************************************************

WTC CAATCAAGTCATTTTGAAGATTCAAATGAGTGTGCCAATCACGCATCTCACGGTGGAATT 5340

MCL CAATCAAGTCATTTTGAAGATTCAAATGAGTGTGCCAATCACGCATCTCACGGTGGAATT 5340

************************************************************

WTC GGACAAACTGTTCAAATGTGGCCGGGTCTTGGTGCAGGGGCTCAATATTTTCACCTTGAT 5400

MCL GGACAAACTGTTCAAATGTGGCCGGGTCTTGGTGCAGGGGCTCAATATTTTCACCTTGAT 5400

************************************************************

WTC AATCAAATCCTTGGTCGAAGATACTCTCATCACGCTCGTCCTCGACGATCATGTTGTGCA 5460

MCL AATCAAATCCTTGGTCGAAGATACTCTCATCACGCTCGTCCTCGACGATCATGTTGTGCA 5460

************************************************************

WTC TGATCAGACAAGCAGTCATCACCTCCCAAAGCTTCCTTTCATCCCATGACAGTGCAGGGT 5520

MCL TGATCAGACAAGCAGTCATCACCTCCCAAAGCTTCCTTTCATCCCATGACAGTGCAGGGT 5520

************************************************************

WTC TTCGAACGATACCCCACCAGGATTGAAGCACACCAAAAGCACGTTCCACATCCTTTCTAA 5580

MCL TTCGAACGATACCCCACCAGGATTGAAGCACACCAAAAGCACGTTCCACATCCTTTCTAA 5580

************************************************************

WTC CACTCTCTTGCATTTGGGCAAACCTCTTTCTCTTCTCACCTTGGGGTTTCGAGATTGTCT 5640

MCL CACTCTCTTGCATTTGGGCAAACCTCTTTCTCTTCTCACCTTGGGGTTTCGAGATTGTCT 5640

************************************************************

WTC TCACAAAAGTTGACCACTGAGGATATATACCATCTGCTAGATAGTATCCTTTGTTGTACT 5700

MCL TCACAAAAGTTGACCACTGAGGATATATACCATCTGCTAGATAGTATCCTTTGTTGTACT 5700

************************************************************

WTC GGTGGCCGTTGATCTCAAAGTTGACAGGTGGGGAGTTTCCTTCTGCAAGCCTCGCAAAGA 5760

MCL GGTGGCCGTTGATCTCAAAGTTGACAGGTGGGGAGTTTCCTTCTGCAAGCCTCGCAAAGA 5760

************************************************************

WTC CTGGAGAACGTTGCAGCACGTTGATATCATTGTGAGAACCTGCCATACCGAAGAAAGAAT 5820

MCL CTGGAGAACGTTGCAGCACGTTGATATCATTGTGAGAACCTGCCATACCGAAGAAAGAAT 5820

************************************************************

WTC GCTATATCCAAAGATCTTGCGATGCCACCGCTTCTAATATGACAGTGCACCCGTTAACAT 5880

MCL GCTATATCCAAAGATCTTGCGATGCCACCGCTTCTAATATGACAGTGCACCCGTTAACAT 5880

************************************************************

WTC GCCCCTTGTACTGGCCCTGCAAAGCAAATGGACAGTTCTTCCACTCCCAATGCATACAAT 5940

MCL GCCCCTTGTACTGGCCCTGCAAAGCAAATGGACAGTTCTTCCACTCCCAATGCATACAAT 5940

************************************************************

WTC CAATGCTGCCAAGCATGCCTGGAAAGCCTCTAGCTGCATTGGTCGCCAACAATCTCTCTG 6000

MCL CAATGCTGCCAAGCATGCCTGGAAAGCCTCTAGCTGCATTGGTCGCCAACAATCTCTCTG 6000

************************************************************

WTC TATCAGCGGCAGTTGGCTGCCTCAAGTATTCTGGGCCAAACACCTCGATCACAGTCTGGC 6060

MCL TATCAGCGGCAGTTGGCTGCCTCAAGTATTCTGGGCCAAACACCTCGATCACAGTCTGGC 6060

************************************************************

WTC AAAACTTGTACATTGACATCAGACATGTTGTCTCACTCAT 6100

MCL AAAACTTGTACATTGACATCAGACATGTTGTCTCACTCAT 6100

****************************************

**Supplementary Table 1** Detailed information of all transcripts obtained by Kallisto and the statistical analysis of transcripts per million (TPM) in wheat chromosomes 3AL, 3B, 3DL and 5BL, both in presence (WT) and in absence (*ph1b* deletion) of the *Ph1* locus. Data represent mean values ± standard error (SE) from RNA samples collected at late leptotene-early zygotene stage in WT and in *ph1b* deletion.

**Supplementary Table 2** Detailed information on the seven EMS mutant lines selected as possessing potentially interesting mutations within *TaZIP4-B2* (Traes_5BL_9663AB85C.1). The two mutant lines (Cadenza1691 and Cadenza0348) which showed reduced number of COs in Cadenza mutant lines are indicated in bold.
